# Thylakoid ultrastructural variations in chlorophyll-deficient wheat: aberrations or structural acclimation?

**DOI:** 10.1007/s00425-024-04362-w

**Published:** 2024-03-13

**Authors:** Elisabetta Aliprandi, Sara Demaria, Andrea Colpo, Marian Brestič, Marek Živčak, Angela Martina, Simonetta Pancaldi, Costanza Baldisserotto, Lorenzo Ferroni

**Affiliations:** 1 Department of Environmental and Prevention Sciences, University of Ferrara, Corso Ercole I D’Este 32, 44121 Ferrara, Italy; 2 Institute of Plant and Environmental Sciences, Faculty of Agrobiology and Food Resources, Slovak University of Agriculture, Trieda A. Hlinku 2, 949 76 Nitra, Slovakia

**Keywords:** Chlorophyll fluorescence, Chloroplast, Grana, Light curve, Photosystem II, Thylakoid, Transmission electron microscopy, *Triticum*

## Abstract

**Main conclusion:**

A structural re-modeling of the thylakoid system, including granum size and regularity, occurs in chlorophyll-deficient wheat mutants affected by photosynthetic membrane over-reduction.

**Abstract:**

In the chloroplast of land plants, the thylakoid system is defined by appressed grana stacks and unstacked stroma lamellae. This study focuses on the variations of the grana organization occurring in outdoor-grown wheat mutants characterized by low chlorophyll content and a tendency for photosynthetic membrane over-reduction. *Triticum aestivum* ANK-32A and *Triticum durum* ANDW-7B were compared to their corresponding WT lines, NS67 and LD222, respectively. Electron micrographs of chloroplasts were used to calculate grana ultrastructural parameters. Photosynthetic parameters were obtained by modulated chlorophyll fluorescence and applying Light Curves (LC) and Rapid Light Curves (RLC) protocols. For each photosynthetic parameter, the difference Δ(RLC–LC) was calculated to evaluate the flexible response to light in the examined lines. In the mutants, fewer and smaller disks formed grana stacks characterized by a marked increase in lateral and cross-sectional irregularity, both negatively correlated with the number of layers per granum. A relationship was found between membrane over-reduction and granum structural irregularity. The possible acclimative significance of a greater proportion of stroma-exposed grana domains in relieving the excess electron pressure on PSI is discussed.

**Supplementary Information:**

The online version contains supplementary material available at 10.1007/s00425-024-04362-w.

## Introduction

Chloroplasts represent a metabolic factory powered by solar radiation that fuels life on Earth (Kirchhoff 2019). The organelle is characterized by the envelope membranes and the thylakoid membrane network. This organization creates three aqueous compartments: the intermembrane space between the two envelope membranes, the stroma between the envelope and the thylakoid membrane, and the thylakoid lumen (Kirchhoff 2019). In land plants and their closest algal relatives, the thylakoid system is a unique membrane network with a bipartite architecture, which consists of appressed grana stacks and unstacked stromal lamellae (Solymosi 2012; Solymosi and Keresztes 2012). Typically, in angiosperms, a granum is formed by 5–25 thylakoid layers with diameters between 300 and 550 nm, and includes core, margins, and end membranes (Mustárdy and Garab 2003; Kirchhoff 2019; Mazur et al. 2021). The grana–intergrana organization is the result of photosynthetic complexes segregation between thylakoid domains, which ensures the highest possible packing density of the grana membranes (Garab 2016). Specifically, the photosystem I (PSI)- light-harvesting complex I supercomplex and the ATP synthase are located in the stroma-exposed thylakoids. Conversely, the photosystem II (PSII) and its light-harvesting complex II (LHCII) are mainly found in the grana core (Miller and Staehelin 1976; Andersson and Anderson 1980; Anderson et al. 1988). The lateral sorting of PSII and PSI depends on the ordered sorting and packing of LHCII-PSII in thylakoid regions that subsequently stack and stabilize the structure of the entire thylakoid system (Garab 2016). Cytochrome *b*_*6*_*f* (Cyt* b*_*6*_*f*) is located in all thylakoid regions, although its distribution can be uneven between stroma-exposed and appressed membranes (Kirchhoff et al. 2017). The ultrastructure of the thylakoid system, also thanks to its dynamism, plays a fundamental role in the functioning of the photosynthetic apparatus in a changing environment (Nevo et al. 2012). For example, swelling of the thylakoid lumen facilitates the diffusion of plastocyanin between Cyt* b*_*6*_*f* and PSI, and thus the activation of electron transport (Kirchhoff et al. 2011). Moreover, the light-acclimated thylakoid architecture is favorable to the lateral movement of LHCII from the grana cores to the grana margins, which promotes the regulation of the excitation distribution between PSII and PSI, as well as a balanced used of linear electron transport from PSII to PSI and cyclic electron transport around PSI (Rantala et al. 2020). At the same time, upon thylakoid swelling, it has been proposed that the photosynthetic membrane is somewhat stretched, which may help an easier diffusion of plastoquinone between PSII and Cyt* b*_*6*_*f* (Gu et al. 2022). The thylakoid ultrastructure is also related to the need for photoprotection of PSII and PSI. PSII activity must be regulated to avoid that an excess of electrons reaches PSI, which is particularly susceptible to over-reduced states (Shimakawa and Miyake 2018). The accumulation of protons in the thylakoid lumen beyond the capacity to use them for the ATP synthesis induces the safe dissipation of the excess absorbed energy into heat, giving rise to the phenomenon called the non-photochemical quenching (NPQ). The cyclic electron flow around PSI participates importantly in the induction of NPQ (Munekage et al. 2002; Nishikawa et al. 2012; Wang et al. 2015; Suorsa et al. 2016; Yamamoto et al. 2016). The thylakoid system, with its special architecture, is the theater where these complex events occur to allow photosynthesis, while preserving the biochemical machinery responsible for light-harvesting and energy conservation. The intimate relation between thylakoid structure and function is not fully understood and is a hot topic in plant cell biology, with methodological contributions deriving from electron microscopy, biochemistry and biophysics (Kirchhoff 2018, 2019; Staehelin and Paolillo 2020; Mazur et al. 2021).

Different studies have already recognized the components of the thylakoid membrane as possible regulators of grana architecture and size (for review, Mazur et al. 2021). A classical approach is the structural grana analysis in mutants characterized by an altered composition of the photosynthetic membranes. Chlorophyll-deficient mutants especially were used as an instructive material to analyze structure–function relations of thylakoids. The current understanding of the assembly of the grana–intergrana architecture (e.g., Garab 2016) suggests that a lower LHCII amount in chlorophyll-depleted mutants should unavoidably lead to smaller grana down to single thylakoids. Differently, empiric evidence from mutants/transformants shows a diversity of structures, ranging from prevailing single thylakoids (Allen et al. 1988; Falbel et al. 1996) to thylakoid doublets (Ferroni et al. 2020), from small grana (Kim et al. 2009; Ma et al. 2017; Friedland et al. 2019) to quasi-WT or unusually large grana (Wang et al. 2018; Nicol et al. 2019; Ferroni et al. 2020). However, such anomalous thylakoid architectures are only seldom analyzed quantitatively, for instance including regularity parameters of the grana stacks. The Granum Lateral Irregularity (GLI) quantifies the irregularity of a granum stack with respect to the uniformity of the thylakoid disk diameters (Kowalewska et al. 2016). The Granum Cross-Sectional Irregularity (GSI) evaluates the shifting of granum membranes in the lateral plane (Mazur et al. 2021).

Durum and bread wheat are separate species (*Triticum durum* and *Triticum aestivum*, respectively), and the latter, hexaploid, was originated from the former, tetraploid, by hybridization with the spontaneous relative species *Aegilops tauschii* (Brenchley et al. 2012). *T. durum* ANDW-7B and *T. aestivum* ANK-32A are mutants characterized by a low chlorophyll content compared to their corresponding wild-type lines, LD222 and NS67, respectively. As in similar cases, their chlorophyll deficiency is attributed to a reduced activity of the Mg chelatase and affects particularly the relative concentration of chlorophyll *b*, which is primarily hosted in LHCII (Falbel et al. 1996; Koval 1997; Watanabe and Koval 2003; Kosuge et al. 2011; Wang et al. 2018; Jiang et al. 2019). Because of the smaller PSII antenna, the mutants show an imbalanced excitation rate of PSI and PSII, which causes a lower PSI/PSII ratio than in the WT lines (Andrews et al. 1995; Terao et al. 1996; Brestič et al. 2015). Moreover, ANDW-7B and ANK-32A are impaired in the cyclic electron transport, which is manifested as a smaller proton motive force and a lower capacity for energy dissipation (Živčak et al. 2019; Ferroni et al. 2020; Colpo et al. 2023a). Very characteristic of such mutants is their tendency to over-reduce the electron transport chain. The regulation of photosynthetic electron flow depends on a combination of many regulatory mechanisms that limit PSI over-reduction (Shimakawa and Miyake 2018). The molecular cause for the defective control of electron transport in ANDW-7B and ANK-32A is unknown and could be linked, e.g., to an altered function of Cyt *b*_6_*f* (Ferroni et al. 2022) or to impaired dynamics of the light-harvesting antennae, as occurs in state transition mutants (Bellafiore et al. 2005; Koskela et al. 2018). Although overall similar with respect to their physiology, under controlled growth chamber conditions ANDW-7B and ANK-32A built aberrant but contrasting thylakoid architectures (Ferroni et al. 2020). ANDW-7B exhibited small grana, single thylakoids, and thylakoid doublets. Conversely, ANK-32A, despite its visibly less abundant thylakoid system, still organized extensive grana stacks, associated with long arrays of single straight and parallel thylakoids (Ferroni et al. 2020). In both cases, the indoor cultivation under a fluctuating light regime resulted in a more severe ultrastructural phenotype (Ferroni et al. 2020).

This study focuses on the variations of the grana–intergrana organization occurring in ANDW-7B and ANK-32A cultivated outdoors. Based on the previous indoor observations (Ferroni et al. 2020), it is conceivable that specific structural traits of the thylakoid system, particularly related to the extent and regularity of the thylakoid stacks, could be associated with specific functional alterations known to characterize the mutants also outdoors (Živčak et al. 2019; Colpo et al. 2023a). To verify such hypothesis, a quantitative grana structure analysis of the light-acclimated samples was carried out in parallel to chlorophyll fluorescence quenching analysis. The response flexibility of the wheat lines to increasing light intensities was probed analyzing comparatively light curves of fluorescence parameters under quasi-steady-state conditions or during a progressive fast increase in irradiance, for which a leaf must rely on prompt regulation mechanisms of the electron flow.

## Materials and methods

### Plant material

The plant material included two chlorophyll-deficient mutant lines of wheat, ANK-32A of *Triticum aestivum* and ANDW-7B of *T. durum*, compared with wild-type NS67 and LD222, respectively. Sowing (30 seeds for each line) took place at the Botanical Garden of the University of Ferrara, Italy, in October 2021. Analyses were performed after the tillering stage in February–April 2022 on the first fully expanded leaf of the main stem. Because cells and chloroplasts present a differentiation gradient along the leaf (e.g., Loudya et al. 2021), sample heterogeneity was reduced by sampling the leaf blade at 5–7 cm from the leaf apex.

### Quantification of photosynthetic pigments

For extraction and quantification of photosynthetic pigments, a segment of ca. 10 mg was cut from 5–6 leaves belonging to as many independent plants per wheat line. The samples were weighed and cut into fragments of about 2 mm^2^, which were immersed in 90% acetone buffered with HEPES–KOH (pH 7.8) and then stored in the dark at − 20 °C for 24 h to allow depigmentation of the samples. Subsequently, the absorbances of the extracts at 470, 647, and 664 nm were evaluated using an Ultrospec 2000 spectrophotometer (Pharmacia Biotech). The concentrations of chlorophyll *a* and chlorophyll *b* were calculated using the equations of Ritchie (2006), and the concentration of carotenoids was approximated using the equation of Wellburn (1994).

### Transmission electron microscopy

For electron microscopy, leaf samples were cut from the leaf blade, excluding the central vein, and choosing randomly three independent plants per wheat line. After rinsing with 0.1 M K–Na phosphate buffer at 4 °C (pH 7.2), the samples were fixed with 3% glutaraldehyde in the same buffer for 4 h at 4 °C (Ferroni et al. 2020). Subsequently, the samples were rinsed repeatedly with the buffer and subjected to 2 h of post-fixation with 1% OsO_4_ at room temperature. The samples were dehydrated in an ascending acetone series and embedded in Durcupan ACM resin, according to routine protocols (Ferroni et al. 2020). Ultrathin sections were contrasted with UranyLess (Electron Microscopy Science) and lead citrate. Finally, they were observed with a Talos L120C electron microscope (ThermoFisher Scientific), operating at 120 kV under transmission mode and equipped with a 16-megapixel Ceta camera (ThermoFisher Scientific).

For ultrastructural morphometrics of grana, at least five chloroplasts contained in as many cells were taken per tested leaf (therefore at least 15 chloroplasts per wheat line), allowing quantitative analysis of at least 130 grana stacks sectioned parallelly to the vertical granum axis per wheat line. This was considered a sufficiently big data set for comparison between samples. The basic ultrastructural parameters were measured with Fiji software (https://imagej.net/software/fiji; Abramoff et al. 2004), using electron micrographs recorded at a magnification of 13.500 × on areas of 90 μm^2^. The morphometric parameters descriptive of the grana structure were calculated according to Mazur et al. (2021) and included: the granum height (*h*), established by measuring the distance between the granum end-membranes; the number of thylakoid layers per granum (*N*); the stacking repeat distance (SRD), which represents the average thylakoid thickness obtained dividing *h* by *N*; the average granum diameter (*d*), which is the mean of all layers forming a granum stack; the granum area in cross section (*Area*); GLI, which is the coefficient of variation (the ratio of the standard deviation to the mean) of layers diameters within the granum; GSI, which is calculated by comparing the granum cross-sectional area and the rectangle area with the same perimeter and height as the granum cross section [(rectangle area– granum area)/rectangle area]. In case GSI was negative, the granum was considered regular and the zero value was assigned. Finally, the sum of GSI and GLI, indicated as GI_TOT_, was used as a synthetic index of total granum irregularity (Colpo et al. 2023b).

### Pulse amplitude modulated chlorophyll a fluorometry

Photochemical responses of PSII were analyzed with a Junior PAM (Walz). Leaves were sampled at 9:00–10:00 AM and immediately dark-acclimated in the laboratory for 15 min on a damp piece of filter paper. After the determination of the minimum fluorescence (*F*_0_), the maximum fluorescence (*F*_M_) was measured by applying a saturating pulse (0.6 s). The variable fluorescence was calculated as *F*_V_ = *F*_M_–*F*_0_ and the maximum quantum yield of PSII as *F*_V_/*F*_M_ = (*F*_M_–*F*_0_)/*F*_M_. For the Light Curve (LC) analysis, the samples were subsequently exposed to actinic light of increasing intensity (65 to 1500 µmol photons m^−2^ s^−1^), each step lasting for 10 min, a time sufficient to allow photosynthetic adjustments. At the end of each interval, the steady-state fluorescence (*F*_T_) and the maximum fluorescence value (*F*_M_′) were determined applying a saturating pulse. For the Rapid Light Curve (RLC) analysis, after the determination of *F*_0_, *F*_M_, and *F*_V_/*F*_M_, the samples are exposed to an actinic light of 285 µmol photons m^−2^ s^−1^ for 15 min to activate the Calvin–Benson–Bassham cycle. At the steady state, RLC was induced through the same nine irradiance intervals used for the LC (65 to 1500 µmol photons m^−2^ s^−1^), each lasting 10 s (White and Critchley 1999; Kalaji et al. 2014). For both LC and RLC, four to six biological replicates were performed for each wheat line.

The fluorescence values were combined to calculate: the actual quantum yield of PSII photochemistry Y(PSII) = (*F*_M_*′*–*F*_T_)/*F*_M_*′* (Genty et al. 1989); the quantum yield of the non-regulatory dissipation Y(NO) = *F*_T_/*F*_M_ (Hendrickson et al. 2004); the quantum yield of the regulatory thermal dissipation Y(NPQ) = 1–Y(PSII)–Y(NO) (Hendrickson et al. 2004); the non-photochemical quenching of chlorophyll fluorescence NPQ = Y(NPQ)/Y(NO) = (*F*_M_–*F*_M_*′*)/*F*_M_*′* (Ferroni et al. 2020).For each parameter, the difference Δ(RLC– LC) was calculated.

### Statistical analyses and correlation matrix

Data obtained from the experiments were analyzed using the Microsoft Office Excel (Microsoft) or Origin^™^ version 2024 (OriginLab). Means comparisons of pigments and fluorometric parameters between mutants and wild-type lines were performed using Student’s *t* test with *α* = 0.05 as the significant threshold. Because the ultrastructural morphometric parameters did not always meet the assumption of normality according to Shapiro–Wilk test, the results are presented as medians with the Q1 and Q3 values, and the pairwise comparison of datasets was done with Wilcoxon signed-rank test with *α* = 0.05 as the significant threshold. The Pearson’s *r* correlation matrix between ultrastructural and photosynthetic parameters was built with Origin^™^ version 2024.

## Results

### Photosynthetic pigment content

A yellow–green phenotype characterized the chlorophyll-deficient wheat mutants, in contrast to the more intense green of WT genotypes. The photosynthetic pigment contents (chlorophylls and carotenoids) confirmed the effects of the genetic defect in mutant lines (Table 1). For both wheat species, the mutants had reduced chlorophyll *a* and chlorophyll *b* levels, but chlorophyll *b* was more affected leading to significantly higher chlorophyll *a/b* ratio in both mutants as compared to the corresponding WT lines. A decrease in the carotenoid content was also found in both mutants, especially in ANK-32A.

**Table 1:** Photosynthetic pigment content of WT and chlorophyll-deficient mutant lines of wheat

	NS67	ANK-32A	LD222	ANDW-7B
Chlorophyll *a* (nmol mg^−1^)	2.49 ± 0.30	1.59 ± 0.17*	1.56 ± 0.36	1.00 ± 0.15
Chlorophyll *b* (nmol mg^−1^)	0.66 ± 0.08	0.30 ± 0.02**	0.43 ± 0.09	0.21 ± 0.03*
Chlorophyll (*a* + *b*) (nmol mg^−1^)	3.15 ± 0.38	1.89 ± 0.19*	1.99 ± 0.45	1.21 ± 0.18
Chlorophyll* a*/*b*	3.80 ± 0.08	5.35 ± 0.23***	3.59 ± 0.07	4.83 ± 0.40*
Carotenoids (nmol mg^−1^)	0.98 ± 0.11	0.65 ± 0.07*	0.70 ± 0.14	0.52 ± 0.09

Mean values ± standard error (*n* = 5–6). Significant differences between mutant and WT are indicated according to Student’s *t* test

*P < 0.05

**P < 0.01

***P < 0.001

### Chloroplast ultrastructure

**Fig. 1:**
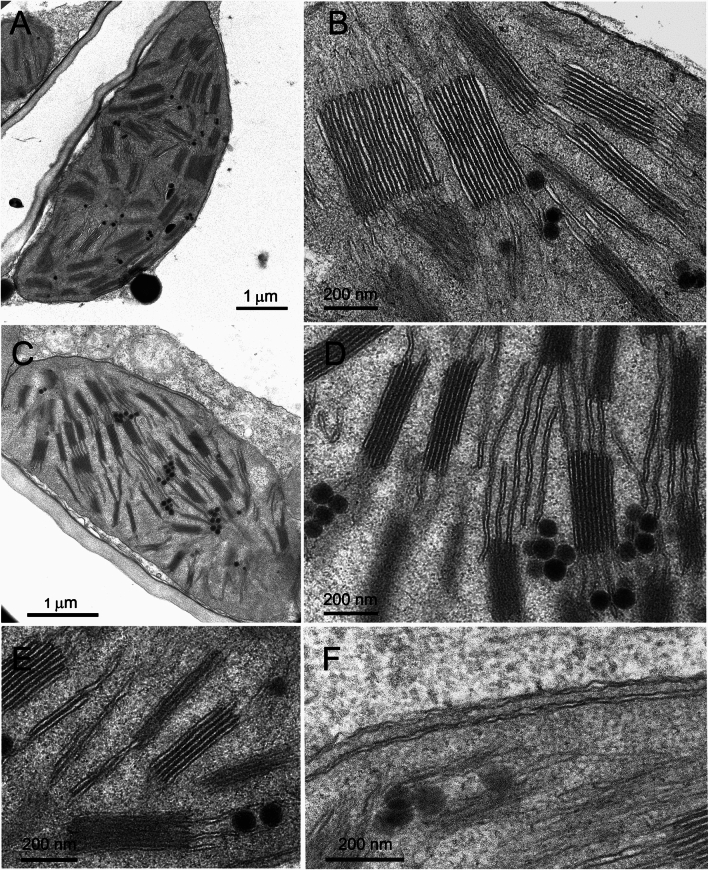
Chloroplast ultrastructure in bread wheat wild type NS67 and chlorophyll-deficient ANK-32A mutant. **A**, **B** A representative chloroplast in NS67 shows an abundant thylakoid system (**A**). Detail of grana stacks and intergrana thylakoids, the latter dilated and with a wavy appearance of their membranes (**B**). **C**–**E** A representative chloroplast in ANK-32A shows a reduced thylakoid system (**C**). Examples of grana and stroma thylakoids with a narrower lumen than in NS67; note some clusters of plastoglobules (**D**). Thylakoid overlaps occurring together with grana stacks (**E**). Detail of a long tubule running parallel to the envelope (**F**)

Bread wheat NS67 presented elliptical chloroplasts characterized by a typical grana–intergrana organization of the thylakoid system and some plastoglobules (Fig. 1A). At higher magnification, the thylakoids showed typical features of active photosynthesis, such as the dilated lumen, the slightly wavy membrane of the intergrana thylakoids, and the slightly swollen grana margins (Fig. 1B). Consistent with the lower chlorophyll content, ANK-32A showed a reduction of the thylakoid system with a less ordered grana–intergrana organization (Fig. 1C). Well-structured grana occurred together with single thylakoids or thylakoids doublets; clusters of plastoglobules were often associated with the thylakoids (Fig. 1D, E). Unlike the WT, common features of active photosynthesis were less evident. In particular, appressed and non-appressed thylakoids had a narrower lumen than the WT (Fig. 1D). A singular feature was a long straight tubule, running under the chloroplast envelope (Fig. 1E).

**Fig. 2:**
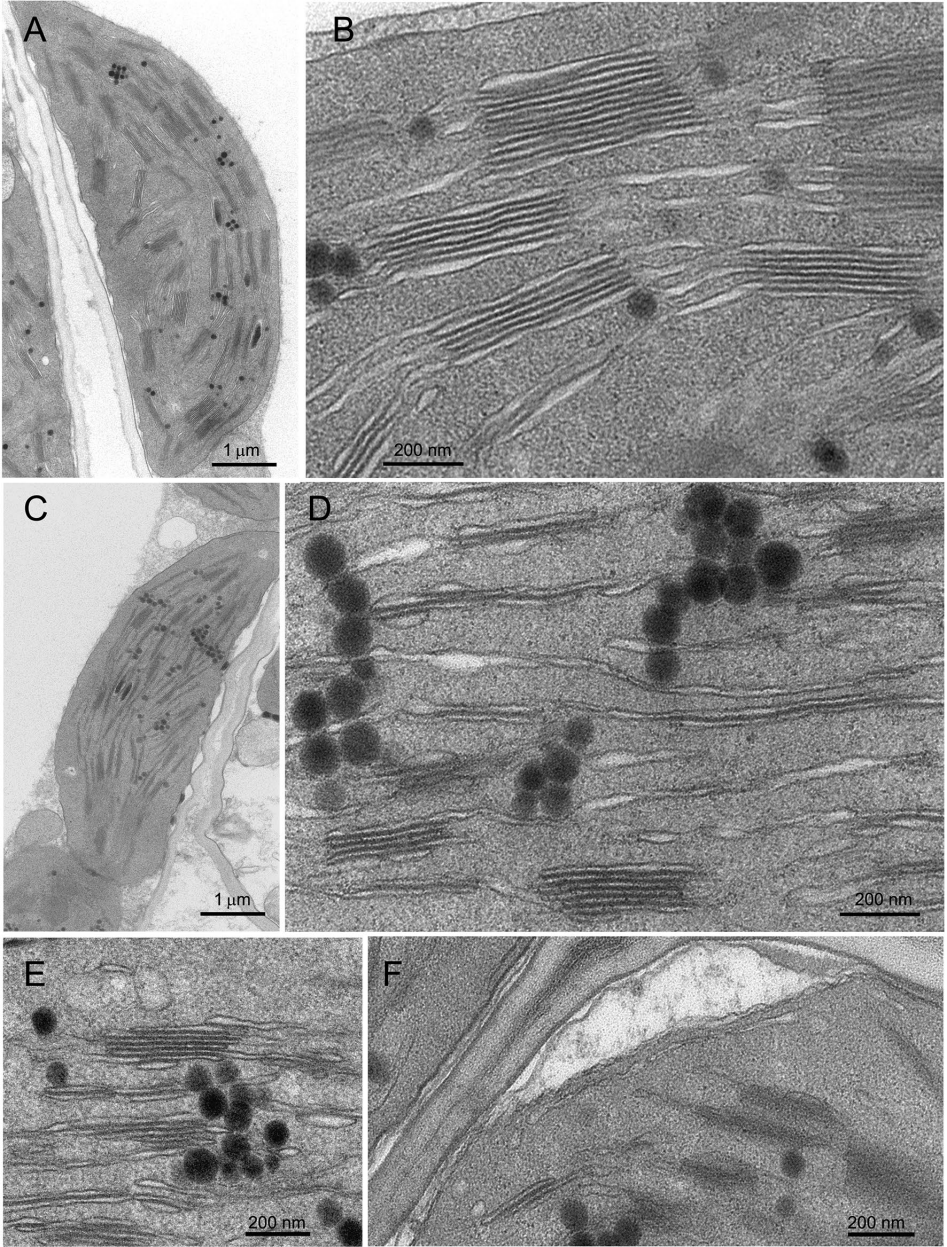
Chloroplast ultrastructure in durum wheat wild type LD222 and chlorophyll-deficient ANDW-7B mutant. **A**, **B** A representative chloroplast with an extensive thylakoid system in LD222 (**A**). Grana stacks and dilated intergrana thylakoids (**B**). **C**–**E** A representative chloroplast in ANDW-7B shows a reduced thylakoid system (**C**). Detail of single thylakoids, thylakoid overlaps and small grana occurring with some clusters of plastoglobules (**D**). Examples of irregular grana associated with a plastoglobule cluster (**E**). A system of tubules and vesicles lays underneath the inner envelope membrane (**F**)

Common characteristics of active photosynthesis occurred in durum wheat LD222, with a typical organization of the thylakoid system (Fig. 2A, B). A strong alteration of the thylakoid architecture characterized the ANDW-7B mutant (Fig. 2C). The grana–intergrana organization was largely replaced by thylakoid doublets or single thylakoids, running almost parallel to each other along the major axis of the organelle (Fig. 2D). As compared to the WT, a less dilated lumen characterized the grana thylakoids, and the stroma lamellae tended to lose their wavy appearance because the thylakoid lumen swelling occurred locally in a discontinuous way (Fig. 2D). Cluster of plastoglobules associated with the thylakoids were typical of ANDW-7B (Fig. 2D, E). Another frequent feature of ANDW-7B chloroplasts was a system of tubules and vesicles laying underneath the inner envelope membrane, which can be referred to as a plastid peripheral reticulum (Fig. 2F).

A morphometric analysis of grana was performed, which in the chlorophyll-deficient mutants was limited to residual grana, defined as stacks of at least three thylakoids. In many cases, the parameters were not distributed normally and, therefore, the medians are reported in Table 2 and the data distribution is shown in Supplementary Figs. S1 and S2.

**Table 2:** Grana ultrastructural parameters of wild-type and chlorophyll-deficient mutants

	*h* (nm)	*N*	SRD (nm)	*d* (nm)	*Area* (nm^2^)	GLI	GSI	GI_TOT_
NS67 (15 chloroplasts, 141 grana)	[104] 134 [186]	[5] 7 [9]	[18.6] 20.2 [22.3]	[388] 457 [538]	[49,200] 70,963 [90,931]	[0.047] 0.069 [0.112]	[0] 0.029 [0.078]	[0.065] 0.120 [0.186]
ANK-32A (18 chloroplasts, 137 grana)	[84] 111 [140]	[4] 6 [7]	[18.3] 19.0 [20.0]	[312] 356 [404]	[32,342] 45,227 [57,027]	[0.070] 0.100 [0.146]	[0.038] 0.074 [0.158]	[0.126] 0.198 [0.273]
*P*	< 10^–4^	< 0.01	< 10^–4^	< 10^–4^	< 10^–4^	< 0.001	< 10^–4^	< 10^–4^
LD222 (18 chloroplasts, 151 grana)	[114] 144 [178]	[5] 6 [8]	[20.8] 22.3 [24.6]	[434] 518 [557]	[56,200] 73,183 [91,596]	[0.031] 0.054 [0.095]	[0.024] 0.062 [0.102]	[0.074] 0.111 [0.190]
ANDW-7B (23 chloroplasts, 155 grana)	[67] 83 [104]	[3] 4 [5]	[18.4] 19.6 [21.1]	[301] 357 [416]	[24,210] 31,940 [41180]	[0.150] 0.245 [0.344]	[0.116] 0.218 [0.308]	[0.280] 0.473 [0.647]
*P*	< 10^–4^	< 10^–4^	< 10^–4^	< 10^–4^	< 10^–4^	< 10^–4^	< 10^–4^	< 10^–4^

*h* Granum height; *N* thylakoid layers per granum; *SRD* stacking repeat distance; *d* granum diameter; *Area* granum area in cross section; *GLI* granum lateral irregularity; *GSI* granum cross-sectional irregularity; *GI*_*TOT*_ total granum irregularity. The number of analyzed chloroplasts and grana is reported for each line. Values are expressed as medians with [Q1] and [Q3]

*P* values were obtained from Wilcoxon signed-ranks test

With respect to the vertical direction of the granum ultrastructure, a significant reduction of the granum height *h* by 17 and 42% affected ANK-32A and ANDW-7B, respectively, as compared with the corresponding WT. The smaller *h* was due to a decreased number *N* of thylakoids per granum and a smaller stacking repeat distance (*SRD*), particularly in ANDW-7B.

In the horizontal direction of the granum, a significant reduction in the average granum diameter *d* occurred in both mutants to a similar extent, by ca. 20–30% shorter than in the WT, and was accompanied by increased granum irregularity. Combined variations in *h* and *d* resulted in completely similar granum *Area* in the two WT lines (*P* = 0.43, Wilcoxon signed-ranks test) and significantly 36 and 56% smaller grana in ANK-32A and ANDW-7B, respectively. The chlorophyll-deficient mutants showed higher GLI values, especially ANDW-7B had 4.5 times higher GLI than LD222. GSI values close to 0 in NS67 indicated an almost cylindrical stack. In LD222 and ANK-32A, the value of GSI was twice as high as in NS67. A 3.5 times in increase in GSI affected ANDW-7B compared to LD222. The two WT lines had the same median synthetic granum irregularity parameter GI_TOT_ of 0.11–0.12 (*P* = 0.63, Wilcoxon signed-ranks test). Both mutants were affected by an increased irregularity than their WT lines, but ANDW-7B especially had 4 times higher GI_TOT_ than LD222.

Collectively, in ANDW-7B, not only the thylakoid system was reduced, but also the granum structure was very much altered with respect to both size and regularity of the stacks as compared to LD222. The same tendency to build smaller grana with quite irregular disks was found in ANK-32A.

### Short-term response of the mutants to increasing irradiance

In a first instance, the physiological uniformity of the plant material with previous experiments carried out outdoors was ascertained by evaluating informative parameters in the dark-acclimated state or the light-acclimated steady state (Colpo et al. 2023a). In Table 3, the values of the maximum quantum yield of PSII photochemistry *F*_V_/*F*_M_ were significantly higher in wheat mutants compared to WT genotypes. Conversely, the values of non-photochemical quenching NPQ were lower in both mutants, especially in ANDW-7B. Consistently, the quantum yield of the regulatory thermal dissipation Y(NPQ) was also reduced in the mutants and accompanied by an increase in the complementary yields of the non-regulatory dissipation as heat and fluorescence Y(NO) and PSII actual photochemistry Y(PSII). Because Y(NO) relates to the reduction state of the plastoquinone pool (Grieco et al. 2012), the increase in Y(NO) confirmed the defective electron flow control in the mutants.

**Table 3:** Photosynthetic parameters measured at the at the end of the light curve analysis at an irradiance of 1500 μmol photons m^−2^ s^−1^

	*F*_V_/*F*_M_	Y(NO)	Y(NPQ)	Y(PSII)	NPQ
NS67	0.788 ± 0.013	0.170 ± 0.008	0.667 ± 0.010	0.163 ± 0.004	3.96 ± 0.22
ANK-32A	0.823 ± 0.003*	0.189 ± 0.004**	0.610 ± 0.009	0.201 ± 0.008*	3.24 ± 0.10*
LD222	0.805 ± 0.002	0.164 ± 0.008	0.660 ± 0.009	0.176 ± 0.008	4.10 ± 0.31
ANDW-7B	0.816 ± 0.004*	0.228 ± 0.005***	0.558 ± 0.012***	0.214 ± 0.011*	2.45 ± 0.09***

Mean values ± standard error (*n* = 5–6). Significant differences between mutant and WT are indicated according to Student’s *t* test

*P < 0.05

**P < 0.01

***P < 0.001

**Fig. 3:**
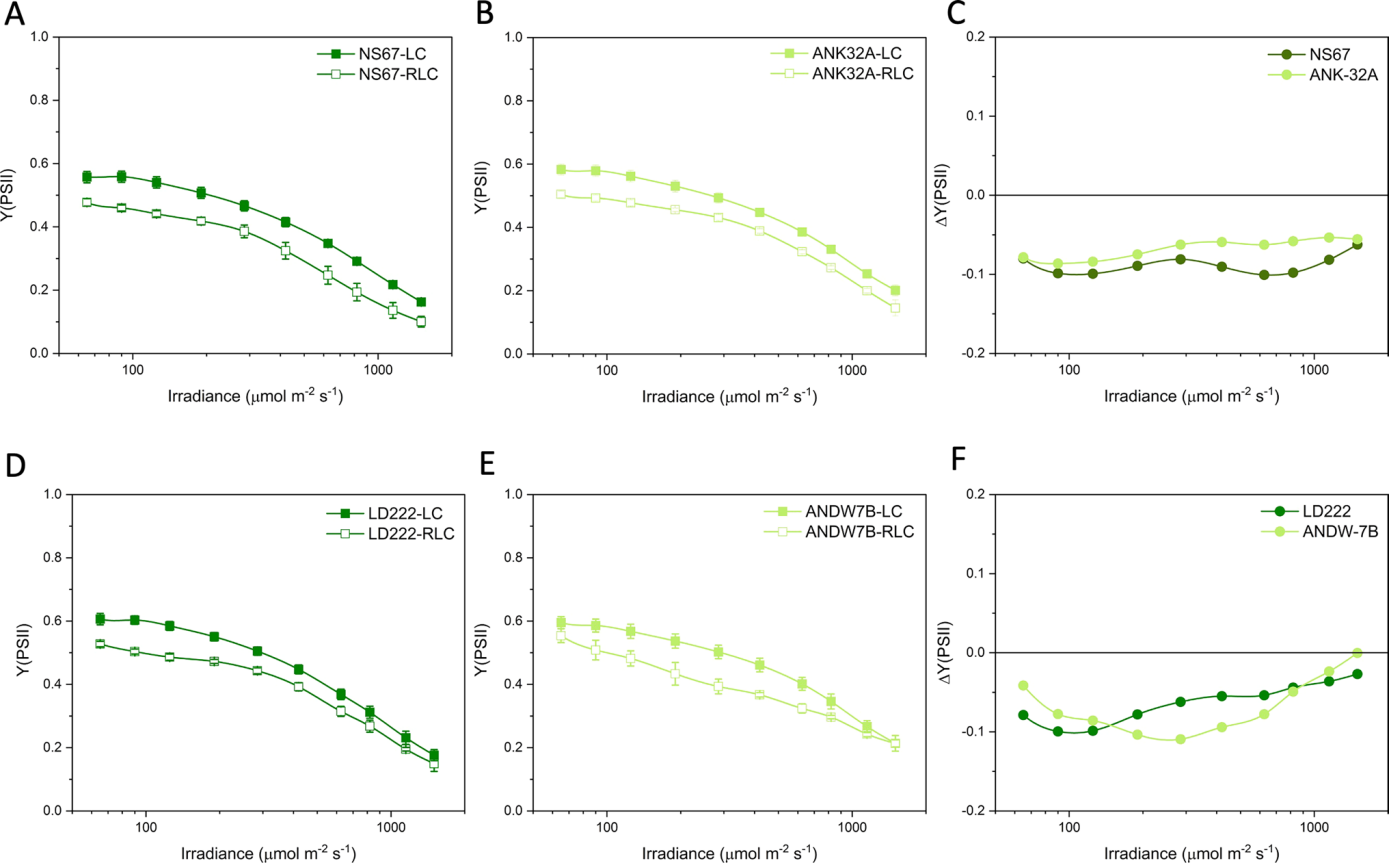
Light response curves of the actual quantum yield of PSII photochemistry Y(PSII) in bread (**A**, **B**) and durum wheat (**D**, **E**). LC steady-state light curve; RLC rapid light curve. Values are means with standard errors of *n* = 4 (RLC) or 5–6 (LC) biological replicates. **C**, **F** ΔY(PSII) is the difference between the mean values obtained with RLC and LC for bread (**C**) and durum wheat lines (**F**)

Expectedly, in all wheat lines, Y(PSII) was lower during the RLC than the LC analysis, mainly because the strength of the electron sinks downstream of the electron transport chain was less effectively activated during a fast rise in irradiance (Fig. 3). Therefore, ΔY(PSII) was negative for all wheat lines, but with divergences between mutants and WT lines (Fig. 3C, F). The major divergence was observed in ANDW-7B compared with LD222, specifically between 200 and 600 μmol photons m^−2^ s^−1^ (Fig. 3F). In contrast, in ANK-32A, ΔY(PSII) was slightly less negative than in NS67 (Fig. 3C).

**Fig. 4:**
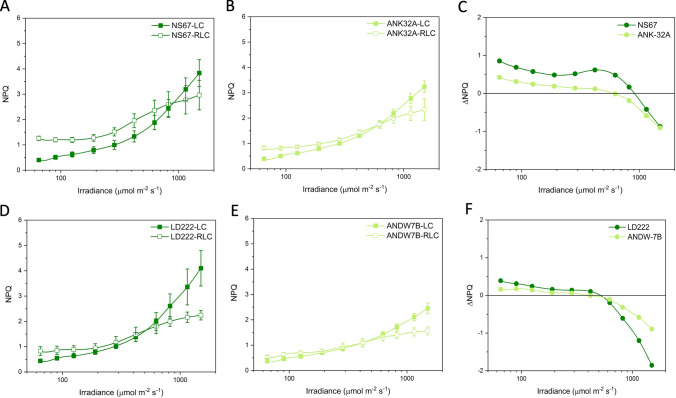
Light response curves of the non-photochemical quenching NPQ in bread (**A**, **B**) and durum wheat (**D**, **E**). LC steady-state light curve; RCL rapid light curve. Values are means with standard errors of *n* = 4 (RLC) or 5–6 (LC) biological replicates. **C**, **F** ΔNPQ is the difference between the mean values obtained with RLC and LC for bread (**C**) and durum wheat lines (**F**)

The light curves of NPQ showed an increasing trend in each genotype, and the mutants were less capable to induce NPQ (Fig. 4). The ability of plants to cope with a fast increase in irradiance depends, among others, on the fast induction of NPQ. ANK32A was less effective than NS67 in inducing NPQ during a fast rise in irradiance and, in both bread wheat lines, the NPQ induction capacity declined above 600 μmol photons m^−2^ s^−1^ (Fig. 4C). In durum wheat, despite the different levels of absolute NPQ induction, ANDW-7B e LD222 had similar ΔNPQ responses to light up to ca. 600 μmol photons m^−2^ s^−1^ (Fig. 4F). The decline in ΔNPQ at higher irradiances was more severe in LD222 than ANDW-7B.

**Fig. 5:**
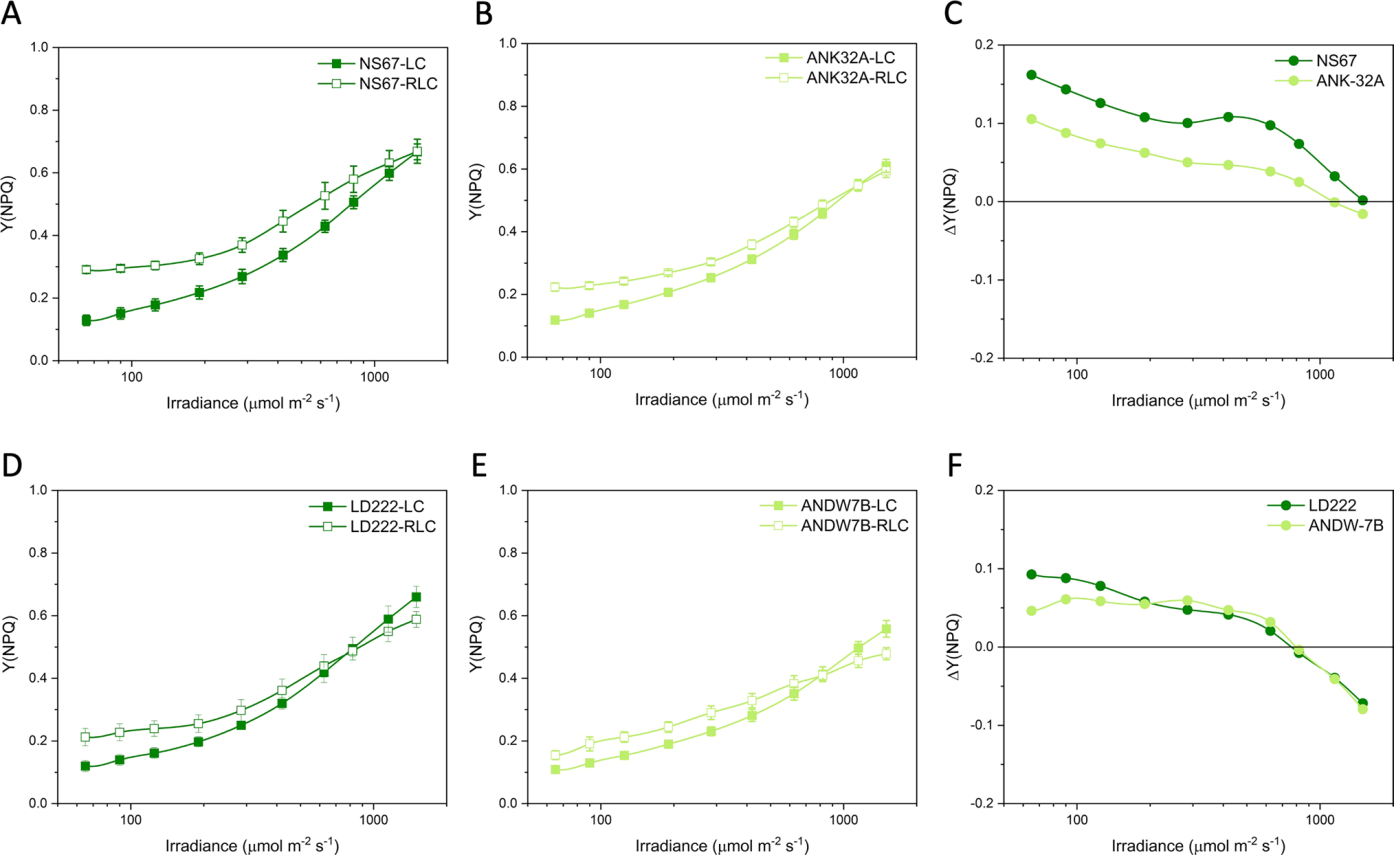
Light response curves of the quantum yield of regulatory thermal dissipation Y (NPQ) in bread (**A**, **B**) and durum wheat (**D**, **E**). LC steady-state light curve; RLC rapid light curve. Values are means with standard errors of *n* = 4 (RLC) or 5–6 (LC) biological replicates. **C**, **F** ΔY(NPQ) is the difference between the mean values obtained with RLC and LC for bread (**C**) and durum wheat lines (**F**)

**Fig. 6:**
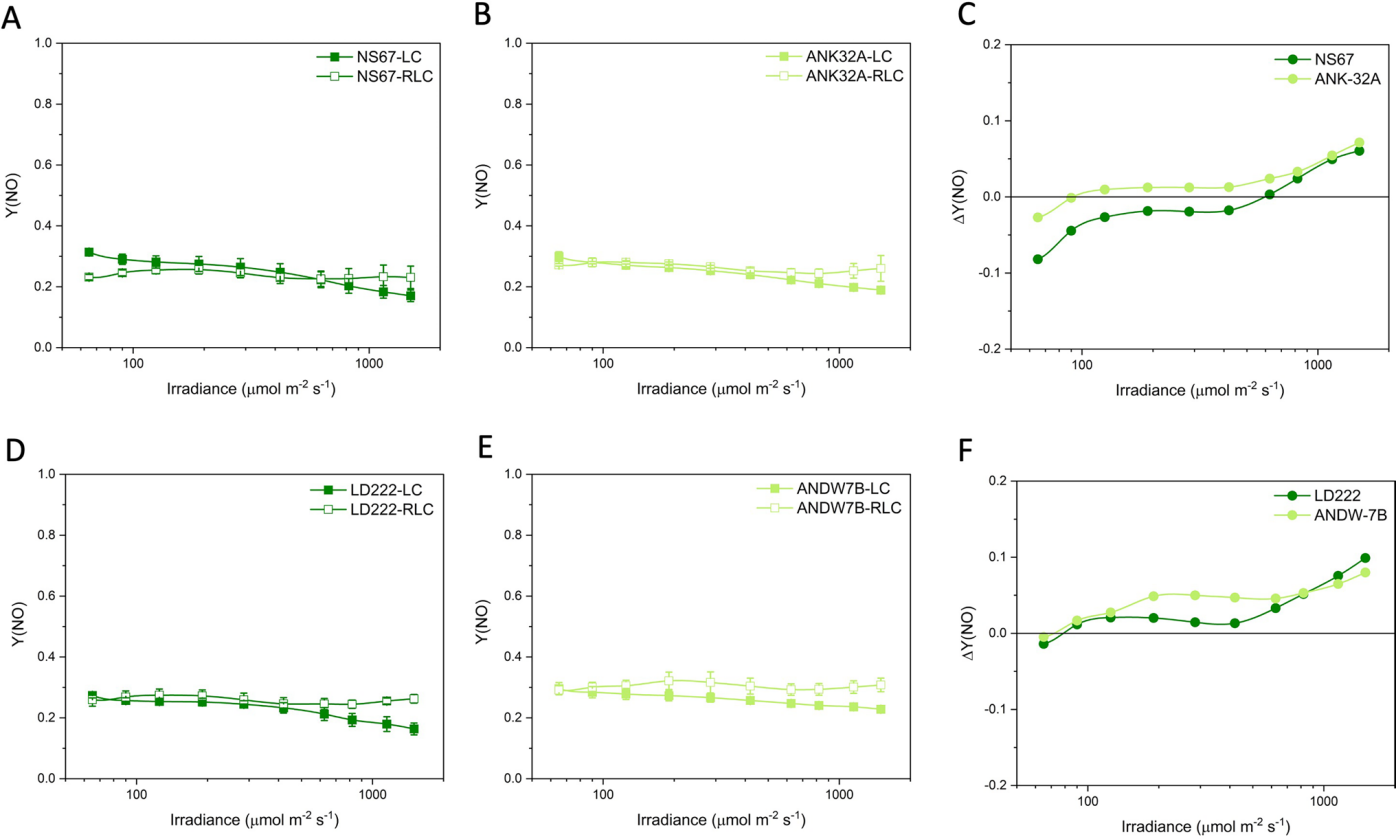
Light response curves of the quantum yield of non-regulatory energy dissipation Y (NO) in bread (**A**, **B**) and durum wheat (**D**, **E**). LC steady-state light curve; RLC rapid light curve. Values are means with standard errors of *n* = 4 (RLC) or 5–6 (LC) biological replicates. **C**, **F** ΔY(NO) is the difference between the mean values obtained with RLC and LC for bread (**C**) and durum wheat lines (**F**)

Positive ΔY(NPQ) in all lines showed the enhanced induction of the regulatory energy dissipation during the RLC (Fig. 5). ANK-32A was less efficient than NS67, while ΔY(NPQ) was almost overlapping between ANDW-7B and LD222 (Fig. 5C–F). The somewhat contrasting response obtained with NPQ and Y(NPQ) indicated that the mutants had differences with respect to Y(NO) (Fig. 6). A well-regulated system should give close-to-zero or even negative ΔY(NO), indicating that the system is effective in preserving the oxidized state of the electron transport chain during a fast rise in irradiance. Such ability was clear in WT lines, particularly in NS67, up to ca. 400 μmol photons m^−2^ s^−1^. When the system was solicited with higher irradiance, ΔY(NO) increased; therefore, the cause for the ΔNPQ increase in the same irradiance interval was not principally a lower capacity for safe heat dissipation, but a lower capacity to control the reduction state of electron carriers. ANK-32A, although less able than NS67 to keep Y(NO) low, in absolute terms interestingly maintained an almost zero ΔY(NO) (Fig. 6C). ANDW-7B was defective in the control of Y(NO) starting from medium–low irradiances (> 100 μmol photons m^−2^ s^−1^) (Fig. 6F).

Therefore, based on ΔY(NO), NS67 was the most flexible wheat line compared with the other genotypes, durum wheat LD222 included. Its mutant ANK-32A was defective in the thermal dissipation induction, but it kept a noticeable ability to control the electron flow upon a fast increase in irradiance. ANDW-7B experienced instead marked problems in electron flow regulation particularly at medium–low irradiance as compared to LD222.

### Linking grana morphometrics and photosynthetic functionality: correlation analysis

**Fig. 7:**
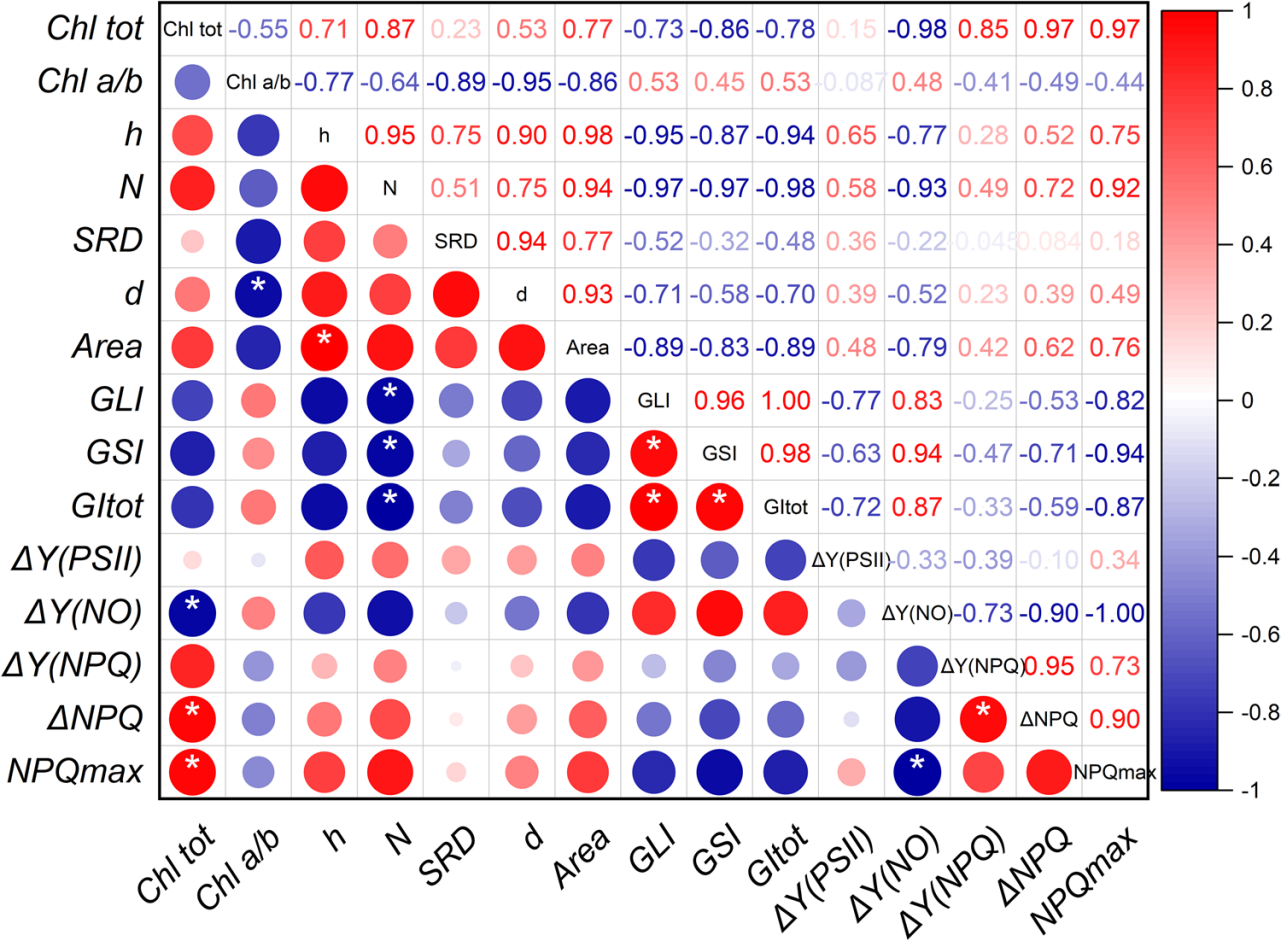
Matrix of Pearson’s *r* correlation coefficients of ultrastructural and photosynthetic parameters in bread and durum wheat lines. The values of Δ(RLC-LC) were sampled at irradiance of ca. 300 μmol photons m^−2^ s^−1^; NPQmax values at 1500 μmol photons m^−2^ s^−1^ at the end of LC. For the chlorophyll and fluorometric parameters, the averages were used, and for the morphometric parameters, the medians, except *N*, for which the average was used. Intense red color marks significant positive correlations, intense blue the negative, according to the scale on the right. The *r* values are also reported and the asterisk marks the statistically significant correlations with *P* < 0.05

A correlation analysis was carried out to verify the presence of significant relations between ultrastructural and photosynthetic parameters in wheat lines, i.e., relations valid across the two species (Fig. 7). The correlation was positive between the total chlorophyll content and the parameters related to the thermal dissipation, both as maximum absolute capacity to induce NPQ (NPQmax) and as dynamic response to a fast rise in irradiance compared to the steady state (ΔNPQ). Accordingly, the correlation was instead negative with the ability to control rapidly the electron transport – ΔY(NO). The ability to control the opening state of PSII upon a fast rise in irradiance – ΔY(PSII)—was not related to the chlorophyll content. No other relationship with the leaf chlorophyll content reached the significance threshold; the closest to significant was the correlation with *N* (*P* = 0.13). The chlorophyll *a/b* ratio correlated positively only with the average granum diameter *d*. Very interesting were the negative correlations linking *N* with the irregularity indexes, as well as GSI and GLI with each other. Finally, the correlations between GSI and NPQmax or ΔY(NO) were very close to the significance level (*P* = 0.056).

## Discussion

Bread wheat NS67 is greener than durum wheat LD222 and, with their respective mutants, they form a graded series of leaf chlorophyll contents: NS67 > ANK-32A ~ LD222 > ANDW-7B (Table 1). Although across wheat lines the leaf chlorophyll content obviously correlates with the ability to keep Y(NO) low, not as much strong correlation was found with structural parameters of the grana (Fig. 7). This result is most probably due to differences intrinsic to the two species, which cause the granum structure to be very similar in NS67 and LD222 despite the lower chlorophyll content in the latter. The two WT lines are likewise similar with respect to the chlorophyll *a/b* ratio, which is instead much higher in the mutants. Chlorophyll *a/b* is common proxy of the PSII antenna size, although it can be influenced by other factors, particularly the PSI/PSII stoichiometry (Lichtenthaler and Babani 2004; Anderson et al. 2012). At least in young wheat mutants, higher chlorophyll *a/b* was shown to correspond to a smaller functional antenna size of PSII (Ferroni et al. 2022; Colpo et al. 2023a). Across wheat lines, an interesting relation links the chlorophyll *a/b* ratio with the average granum diameter *d* (Fig. 7). In angiosperms, *d* is almost invariably of ca. 500 nm independent of species and growth conditions, probably fixed evolutionarily to limit constraints in the diffusion of mobile electron carriers (Anderson et al. 2008; Höhner et al. 2020; Colpo et al. 2023b). In wheat mutants, the association between high chlorophyll *a/b* and reduced *d* at about 350 nm could result from lower LHCII availability and consequently altered PSII-LHCII sorting during grana formation (see Garab 2016). However, in the mutants, the grana are not just downsized stacks formed by a lower number of smaller disks, but within each granum, the layers occur in much variable diameters and tend to slide apart from each other. GLI and GSI catch such complementary aspects of the granum morphology and allow a quantification of the granum irregularity (Kowalewska et al. 2016; Mazur et al. 2021). A significant, strong increase in granum irregularity, comprehensively measured by GI_TOT_, is a characterizing trait of the mutants and is negatively proportional to the number of disks per granum (Table 2, Fig. 7). Especially the negative relationship between GSI and NPQmax can provide a clue to the almost completely unknown relevance of the grana structural irregularity for the functional adjustment of the thylakoid system (Mazur et al. 2021).

Upon illumination, thylakoids swell because of the osmotic water flux from the stroma to the lumen following the lumen acidification (Li et al. 2020). Chlorophyll-deficient wheat is typically impaired in cyclic electron flow around PSI, leading to a lower capacity to accumulate protons in the lumen and consequently to lower NPQ induction (Brestič et al. 2015; Živčak et al. 2019; Ferroni et al. 2020). In the resulting less dilated thylakoids (or smaller stacking repeat distance, SRD), the appressed membranes are less stretched, and the electron flow can be further impeded by slower diffusion of plastoquinone (see Gu et al. 2022). On one hand, the irregular grana seen in the mutants might simply be a consequence of weaker PSII-LHCII interactions to keep the stacks intact. On the other hand, increasing the proportion of the grana margins and end membranes, as was obtained by enhancing GLI and GSI*,* respectively (Mazur et al. 2021), could allow some relief from the over-charge of the intersystem electron carriers. Grana margins are the domains most directly and dynamically engaged in the regulation of the electron flow (Rantala et al. 2020), particularly allowing favorable PSII–PSI interactions for excitation distribution and photoprotective energy spillover from PSII to PSI (Grieco et al. 2015; Ifuku 2023). Much less clear is the functional meaning of an increase in the end granal membranes. It has been hypothesized that extended end membranes improve the efficiency of the proton circuit for ATP synthesis (Anderson et al. 2012), but they could also reflect a sub-localization of Cyt*b*_*6*_*f* that enhance the cyclic electron flow capacity (see Tikhonov 2023). Therefore, in chlorophyll-deficient wheat, the enhanced granum irregularity can have an acclimative significance, possibly further helped by an easier diffusion of mobile electron carriers allowed by reduced *d* (Höhner et al. 2020) and supported by the enlarged acceptor pool size downstream of PSI (Ferroni et al. 2022). When the shifting of thylakoid membrane in the granum lateral plane is brought to its extreme, a thylakoid overlap (doublet) is probably the remnant of what cannot be defined a granum anymore (Mazur et al. 2021; Figs. 1E, 2D).

An interesting consequence of the metabolic cost paid by the mutants to regulate the thylakoid system is the occurrence of the plastid peripheral reticulum in the shape of vesicles and tubules (Lindquist et al. 2016; Figs. 1F, 2F). Although the reticulum is part of the normal plastid metabolism, it is more easily observed when the chloroplast has an increased transport demand of metabolites to maintain the inner membranes (Lindquist et al. 2016). Functional and structural maintenance of the thylakoid system in mutants requires very likely an extra biosynthetic effort by the plant and, ultimately, diverts resources away from growth and reproduction (Colpo et al. 2023a).

In conclusion, in chlorophyll-deficient wheat cultivated outdoors under natural conditions, the thylakoid system is reorganized to build smaller grana in the vertical (*N*) and horizontal direction (*d*). The decrease in the number of layers forming a stack is linked to the tendency to over-reduce the photosynthetic membrane upon a sudden rise in irradiance, but also to an increased level of granum structural irregularity. Given the regulatory relevance currently assigned to the stroma-exposed domains of grana, it is possible that the enhanced irregularity may have an acclimative significance in relieving the excess electron pressure on PSI.

### Supplementary Information

Below is the link to the electronic supplementary material.

Supplementary file1 (DOCX 28228 KB)

## Data Availability

The datasets generated and analyzed during the current study are available from the corresponding author on reasonable request.

## Abbreviations

*d*: Average granum diameter
*F*_0_: Minimum chlorophyll *a* fluorescence in the dark-acclimated state
*F*_M_: Maximum chlorophyll *a* fluorescence in the dark-acclimated state
*F*_M_′: Maximum chlorophyll *a* fluorescence in the light-acclimated state
*F*_T_: Steady-state chlorophyll *a* fluorescence
*F*_V_: Variable chlorophyll *a* fluorescence
GI_TOT_: Total granum irregularity
GLI: Granum lateral irregularity
GSI: Granum cross-sectional irregularity
*h*: Granum height
LC: Light curve of chlorophyll fluorescence parameters
*N*: Number of thylakoids per granum
NPQ: Non-photochemical quenching
RLC: Rapid light curve of chlorophyll fluorescence parameters
Y(NO): Quantum yield of the non-regulatory energy dissipation
Y(NPQ): Quantum yield of the regulatory energy dissipation
Y(PSII): Quantum yield of PSII photochemistry
