# Supplementary material for: Thylakoid ultrastructural variations in chlorophyll-deficient wheat: aberrations or structural acclimation?
Source: Planta. 2024 Mar 13;259(4):90. doi: 10.1007/s00425-024-04362-w (PMC10937782; doi:10.1007/s00425-024-04362-w)
Supplement: Supplementary file 1 — Supplementary file1 (DOCX 28228 KB) [file 425_2024_4362_MOESM1_ESM.docx]

**Thylakoid ultrastructural variations in chlorophyll-deficient wheat: aberrations or structural acclimation?**

Elisabetta Aliprandi, Sara Demaria, Andrea Colpo, Marian Brestič, Marek Živčak, Angela Martina, Simonetta Pancaldi, Costanza Baldisserotto, Lorenzo Ferroni

**Supplementary Figure S1**

**
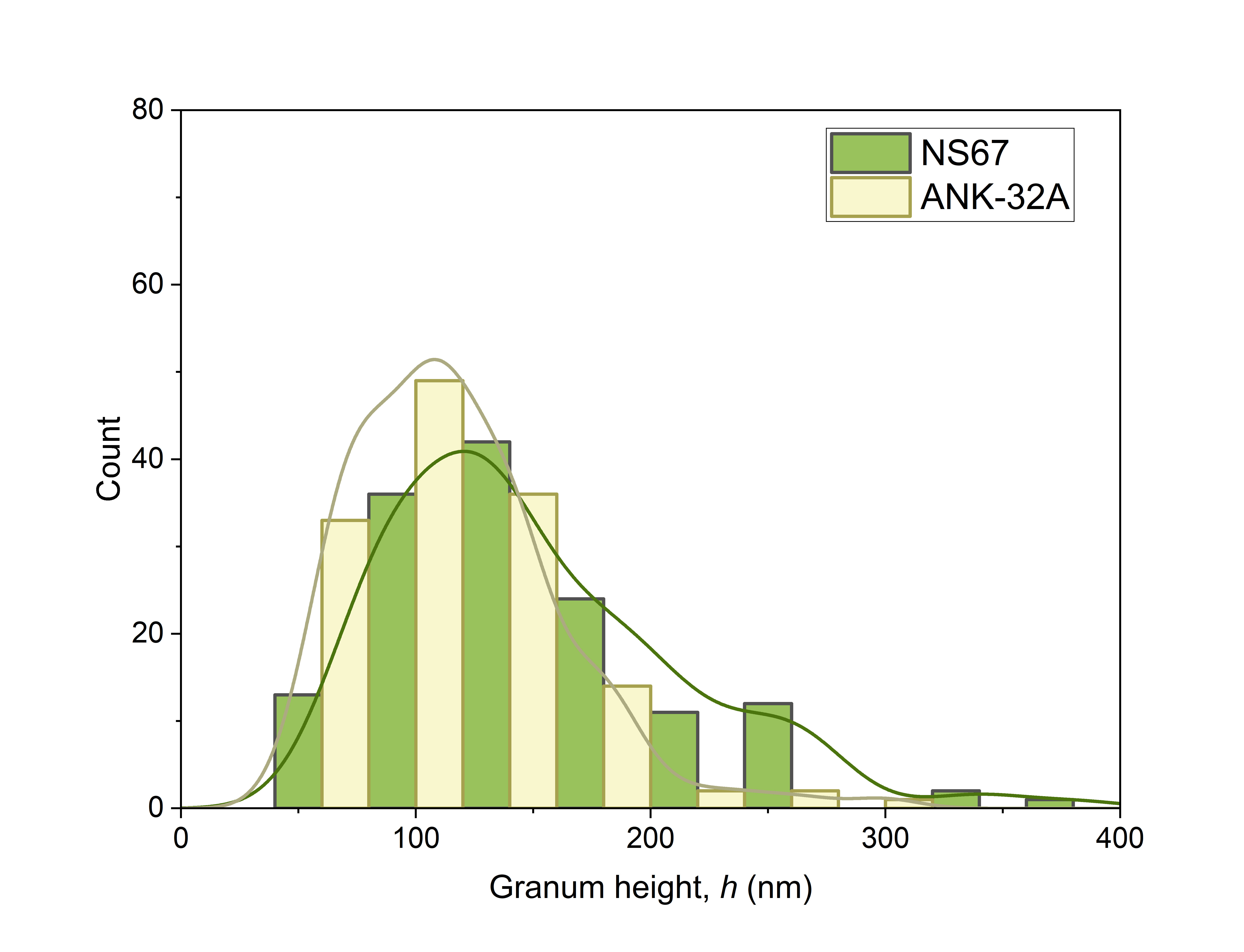
** **
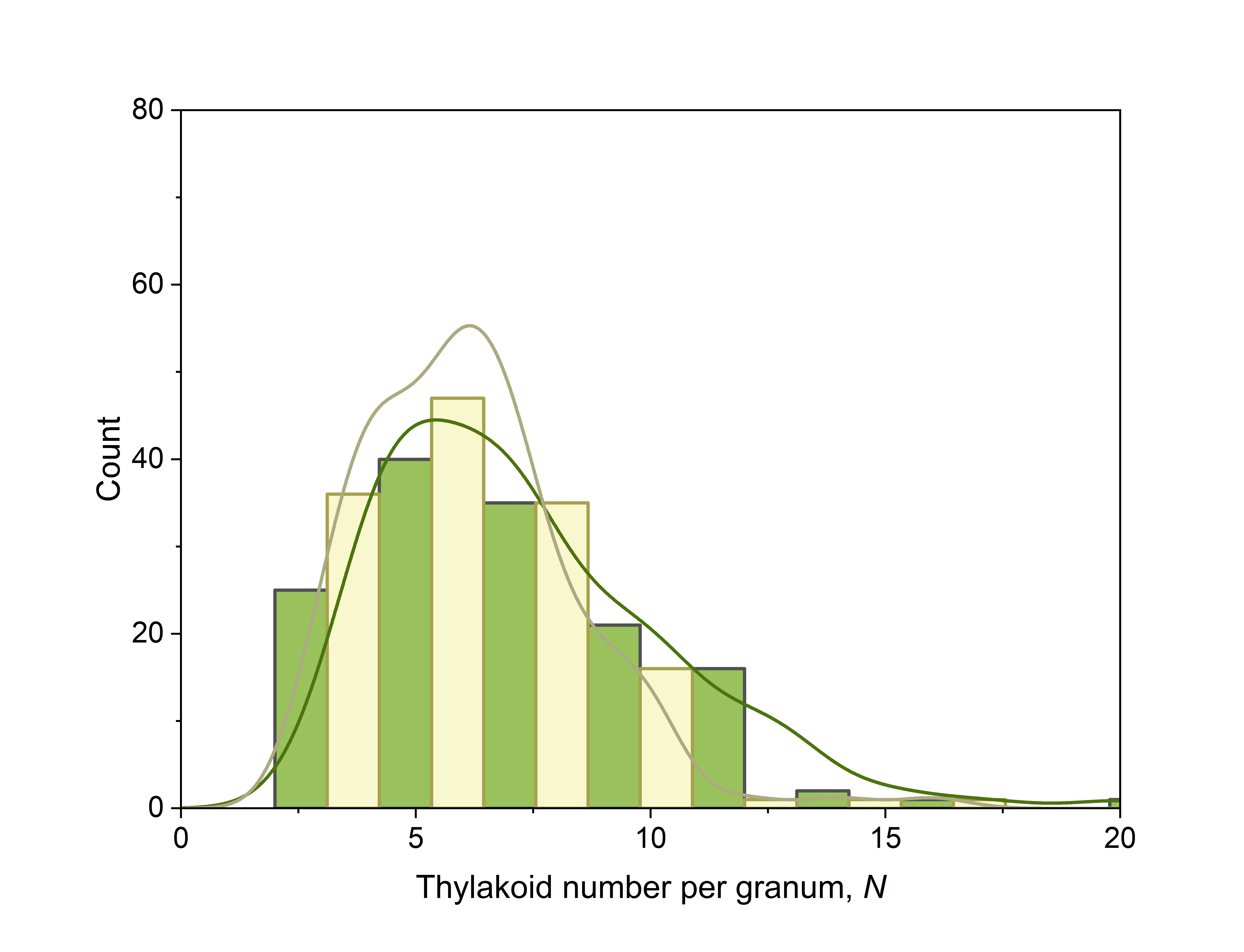
**
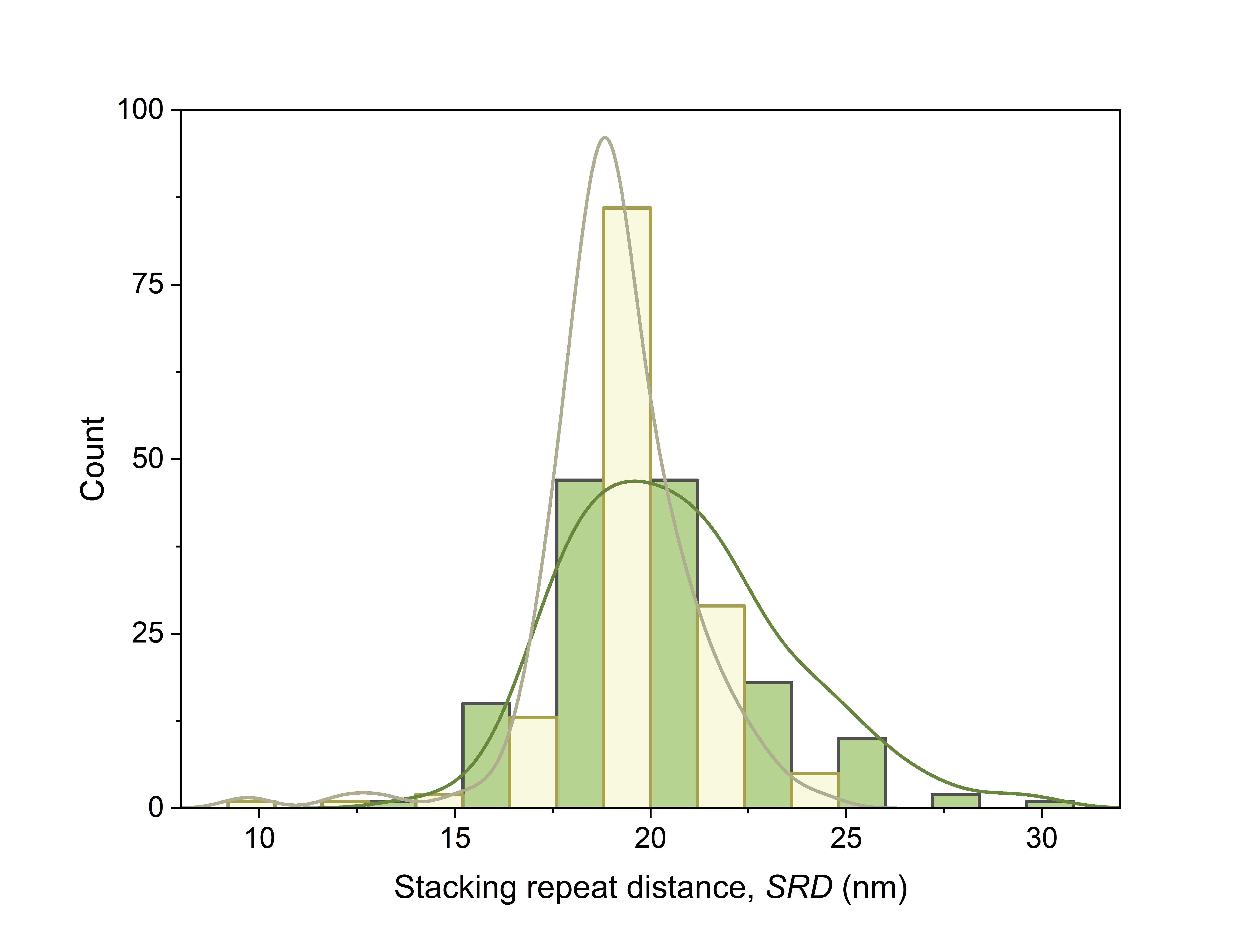


**
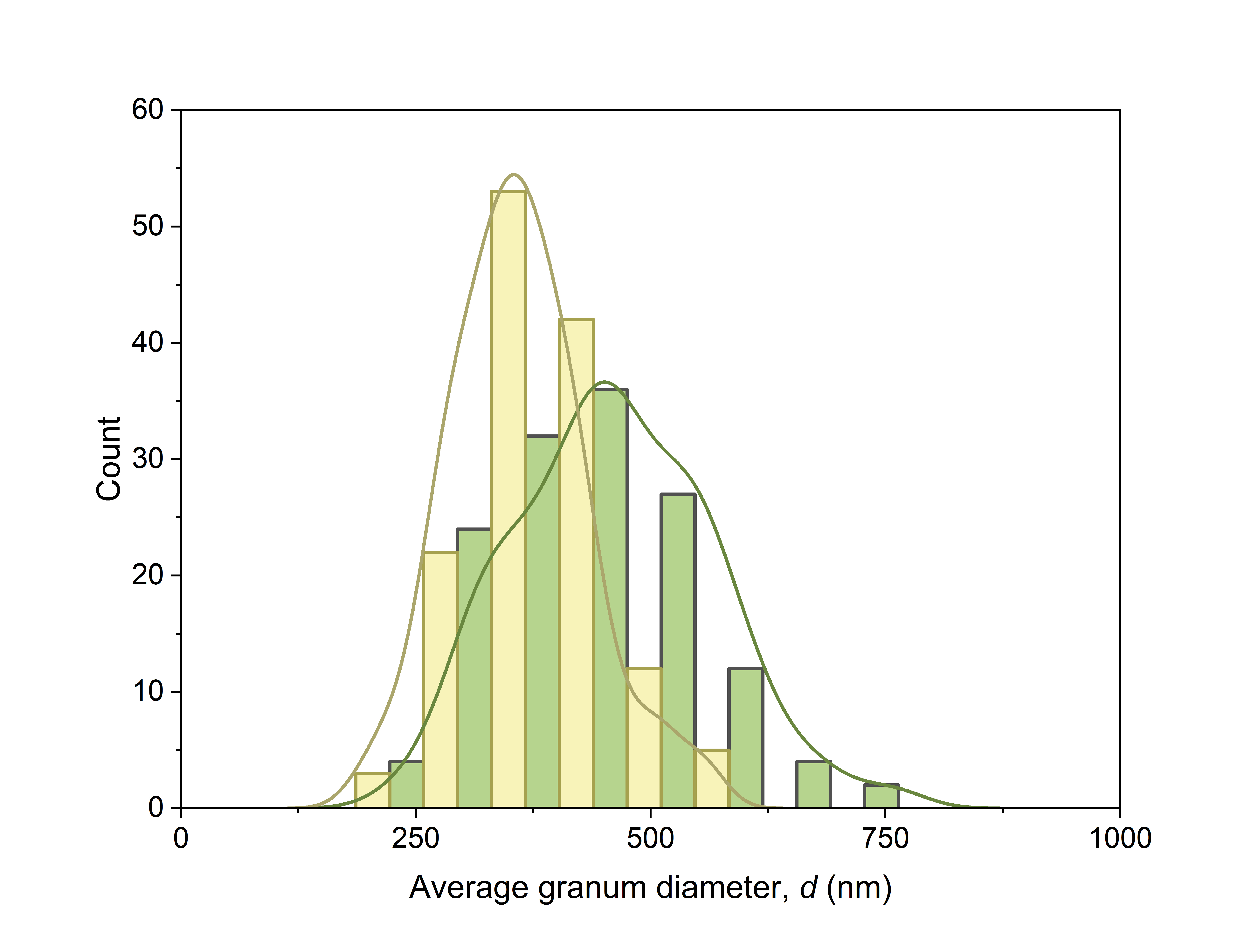

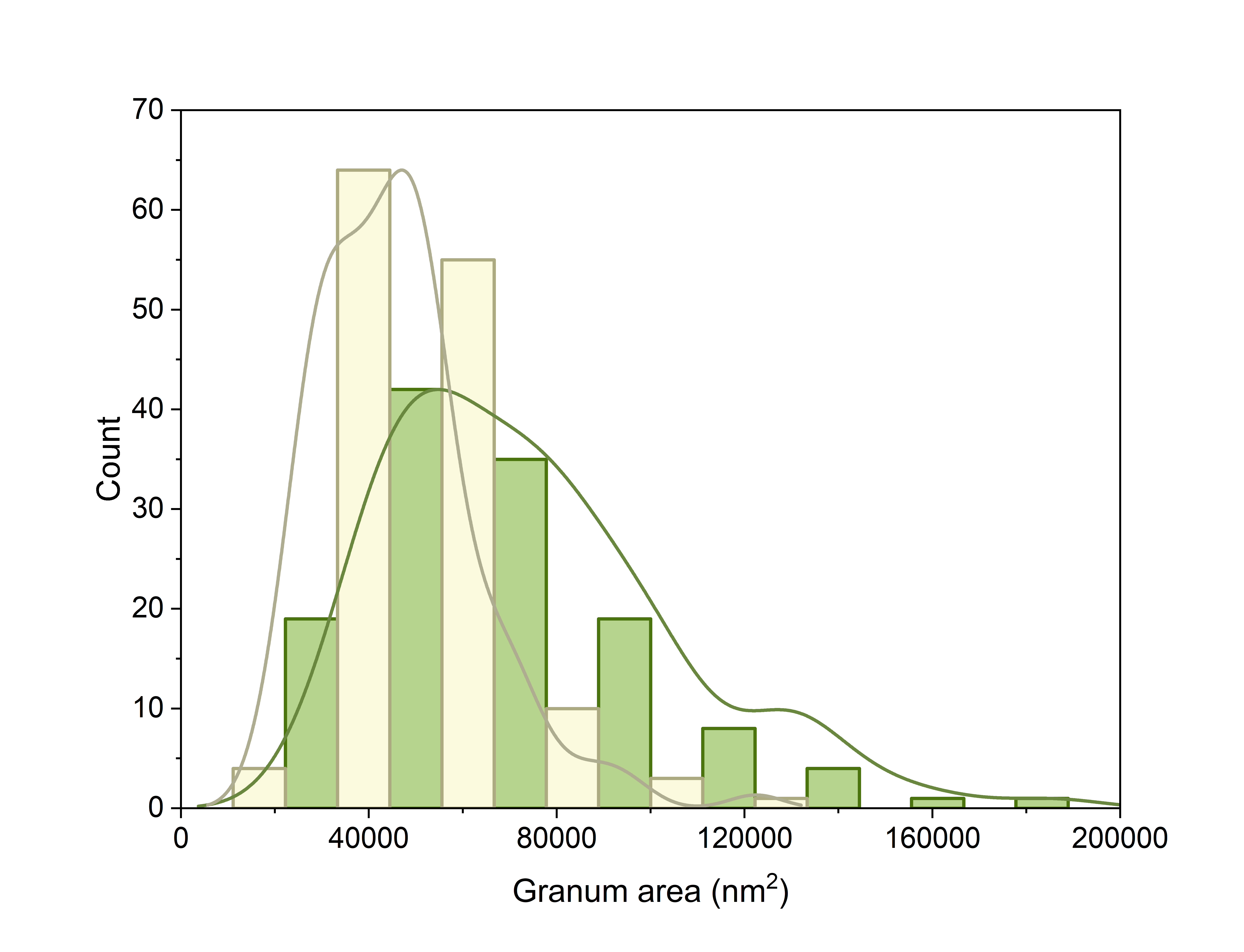
**

**
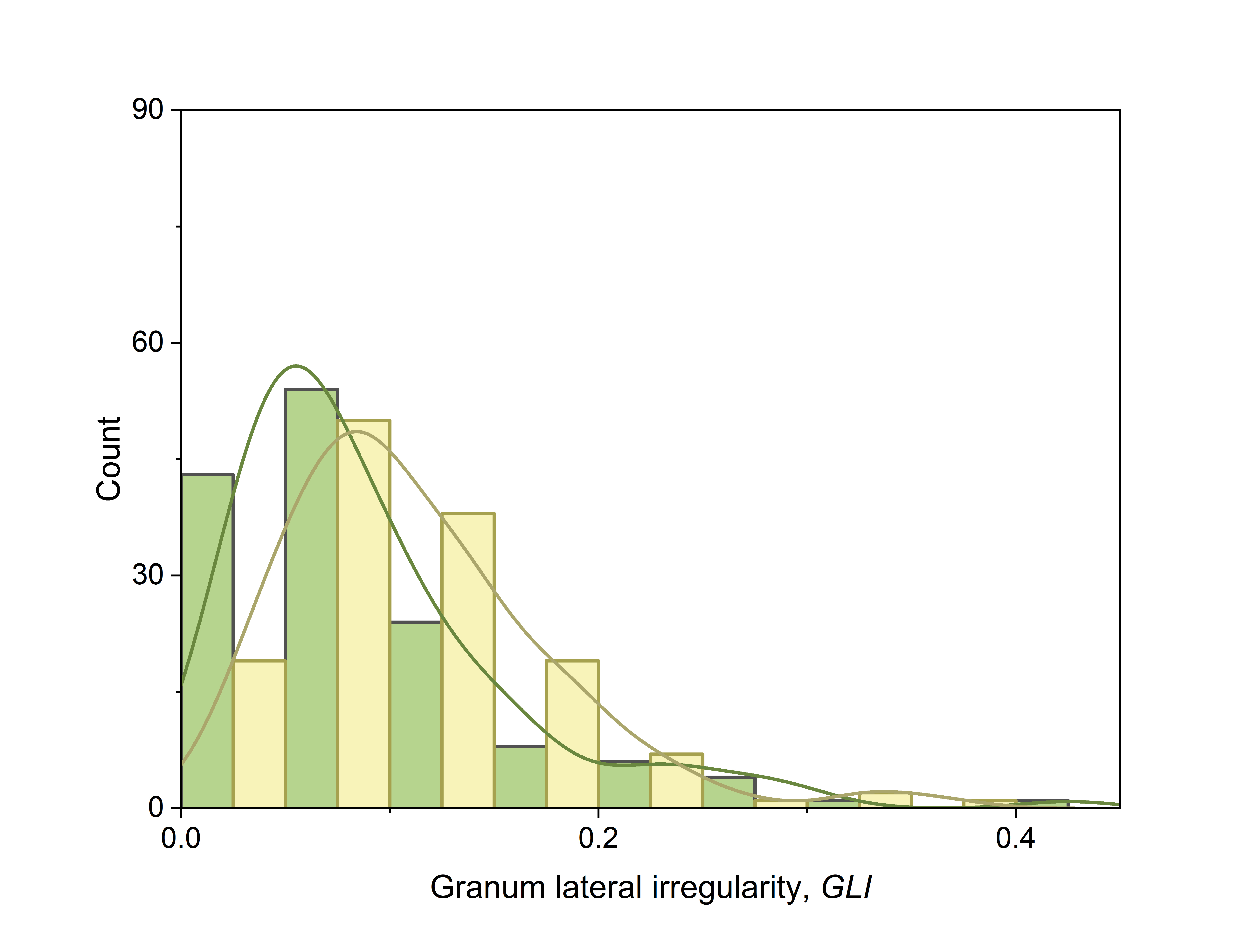
** **
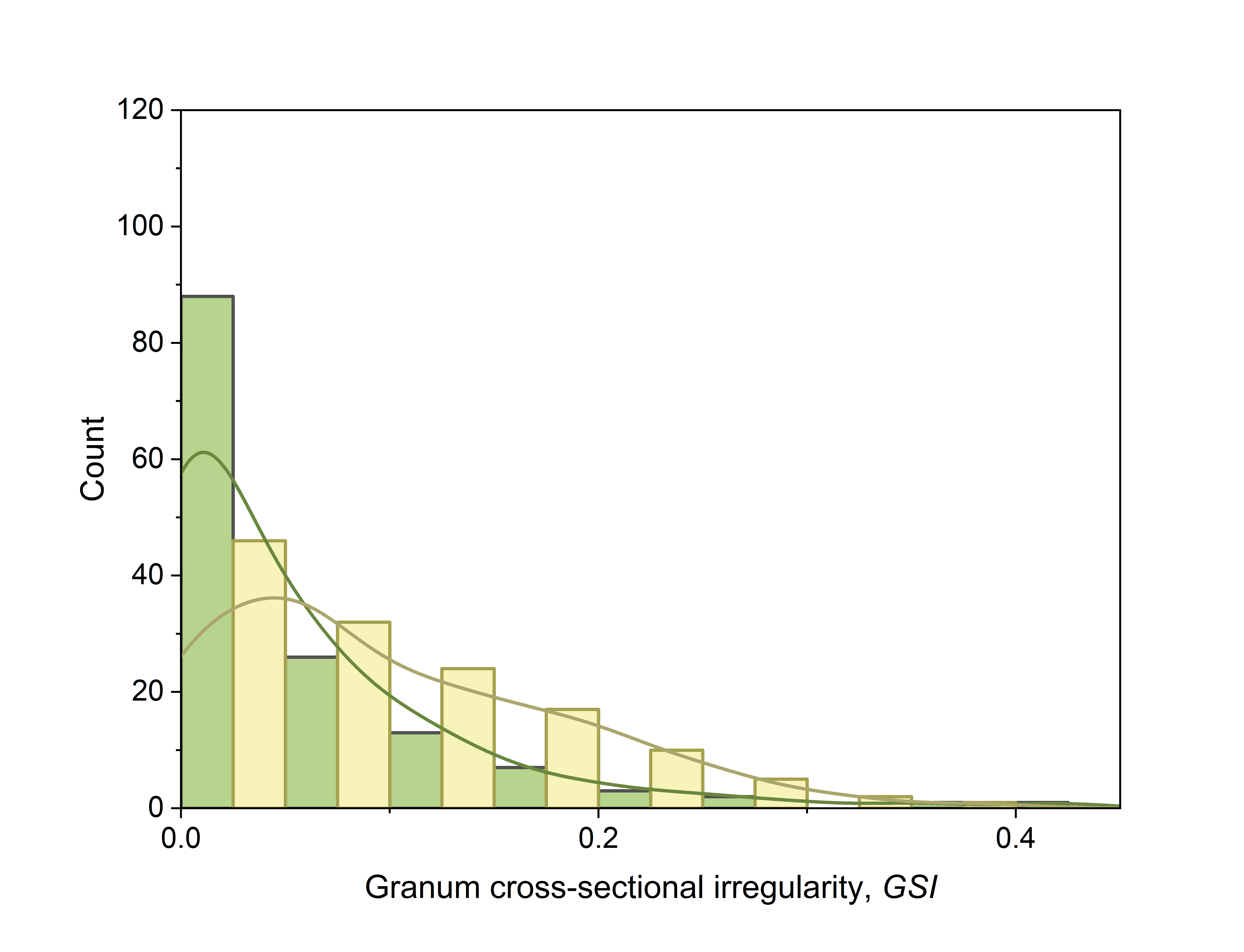
**
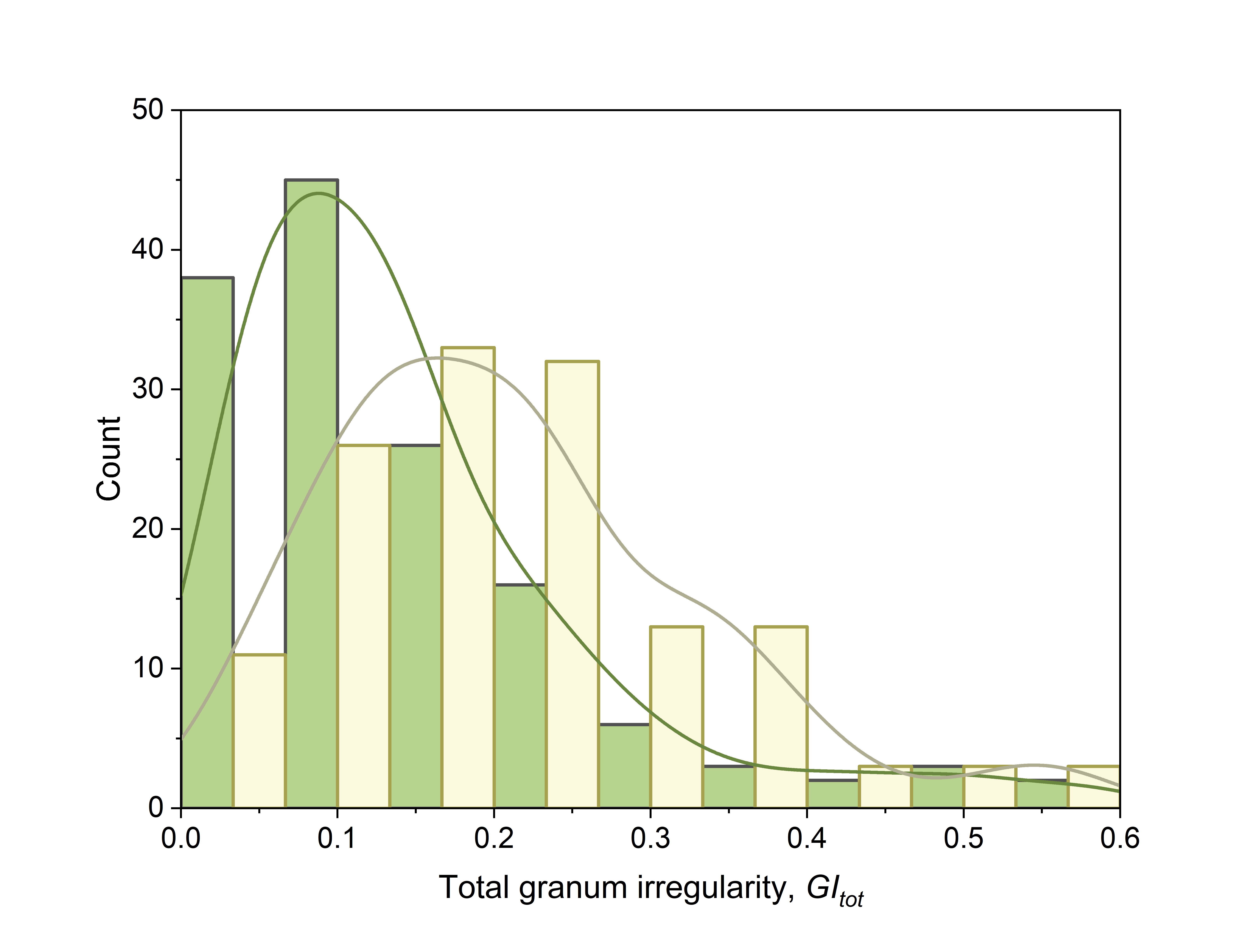


Distribution of the morphometric parameters calculated for the grana stacks in bread wheat NS67 and its chlorophyll-deficient mutant ANK-32A. The samples size was of 141 and 137 grana, respectively. For the definition of the parameters, see the main text. In the graphs, the data distribution was approximated using the “kernel smooth” function of Origin Pro version 2024. In all cases, except *d* in both wheat lines, the hypothesis of normality was rejected based on Shapiro-Wilk test.

**Supplementary Figure S2**


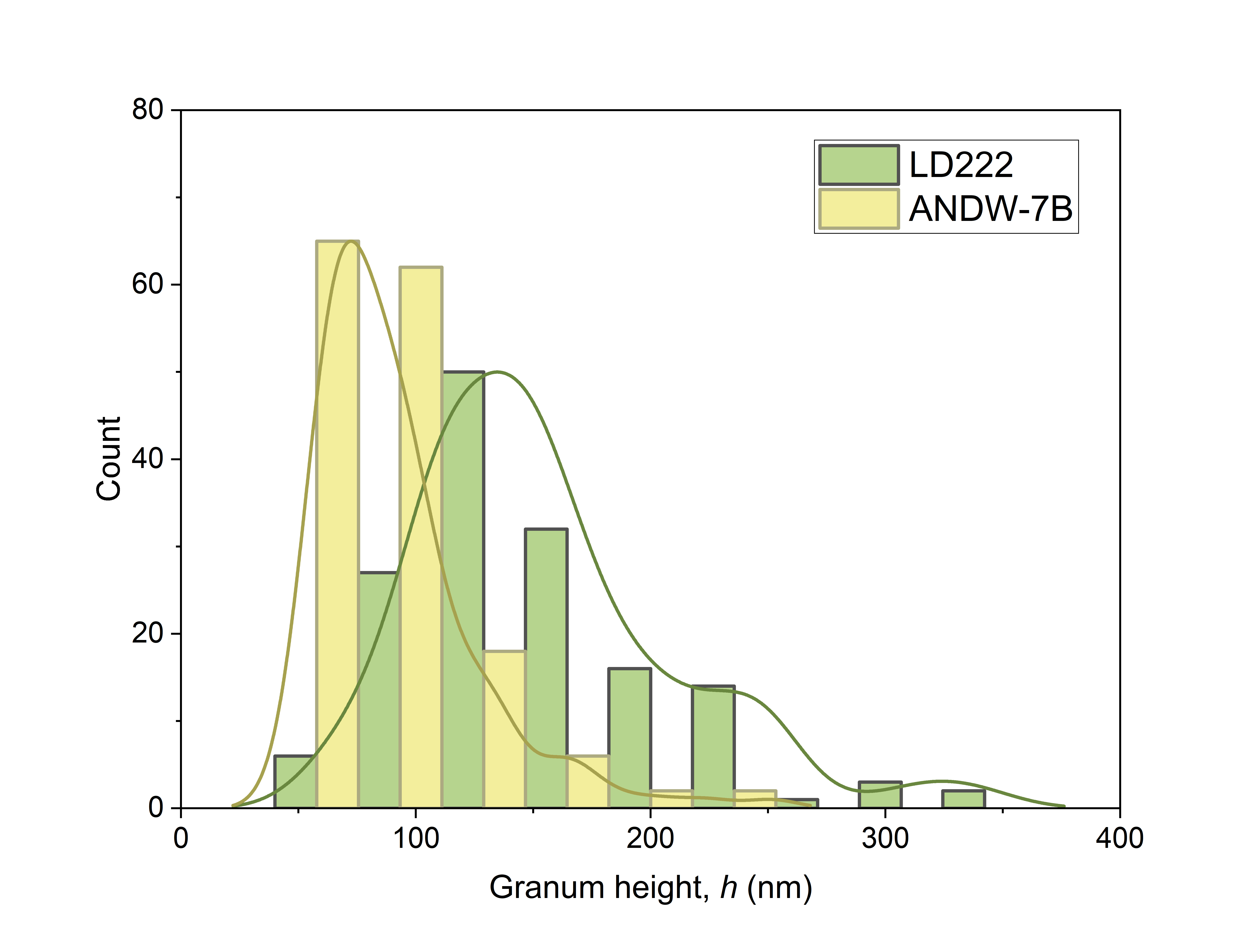

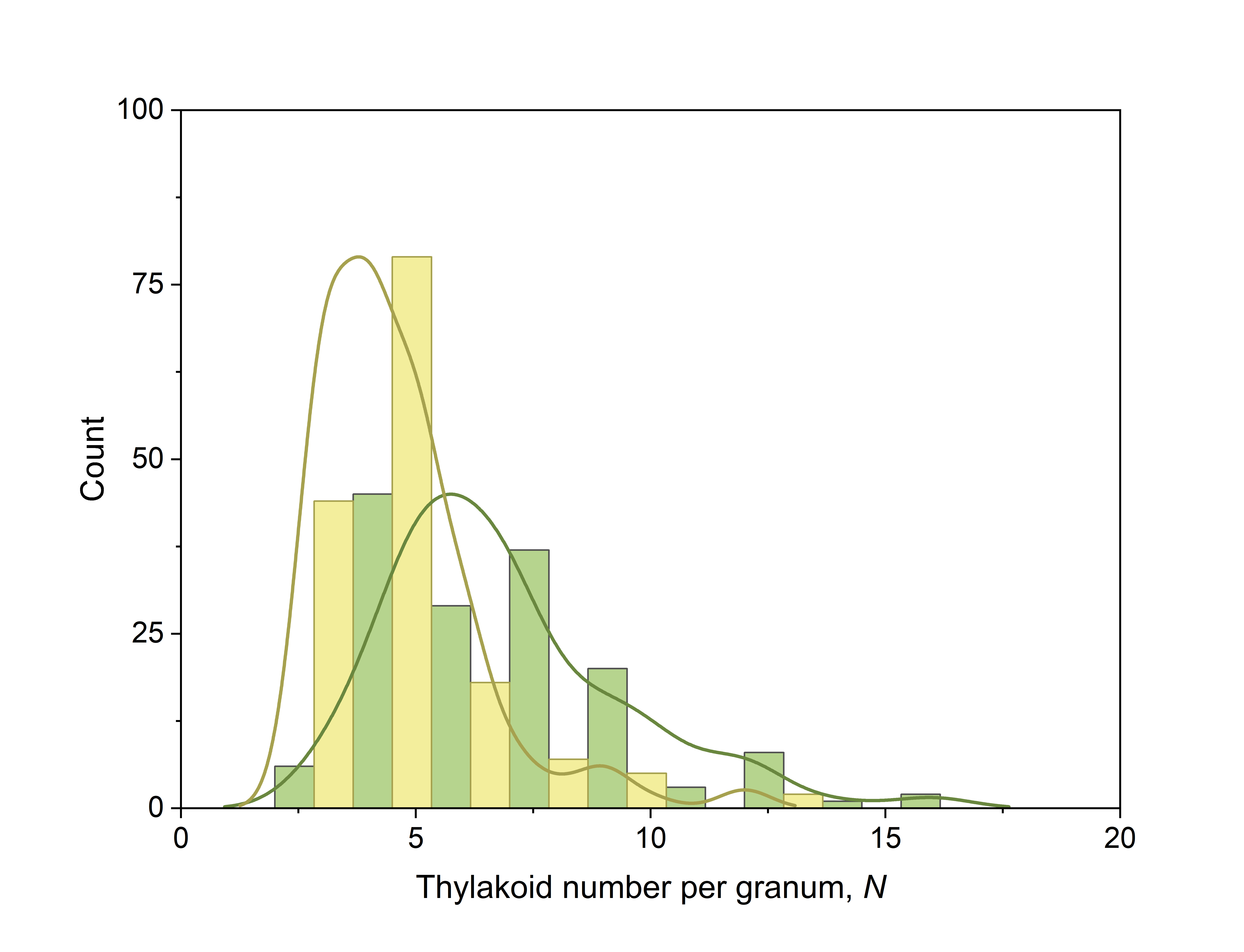

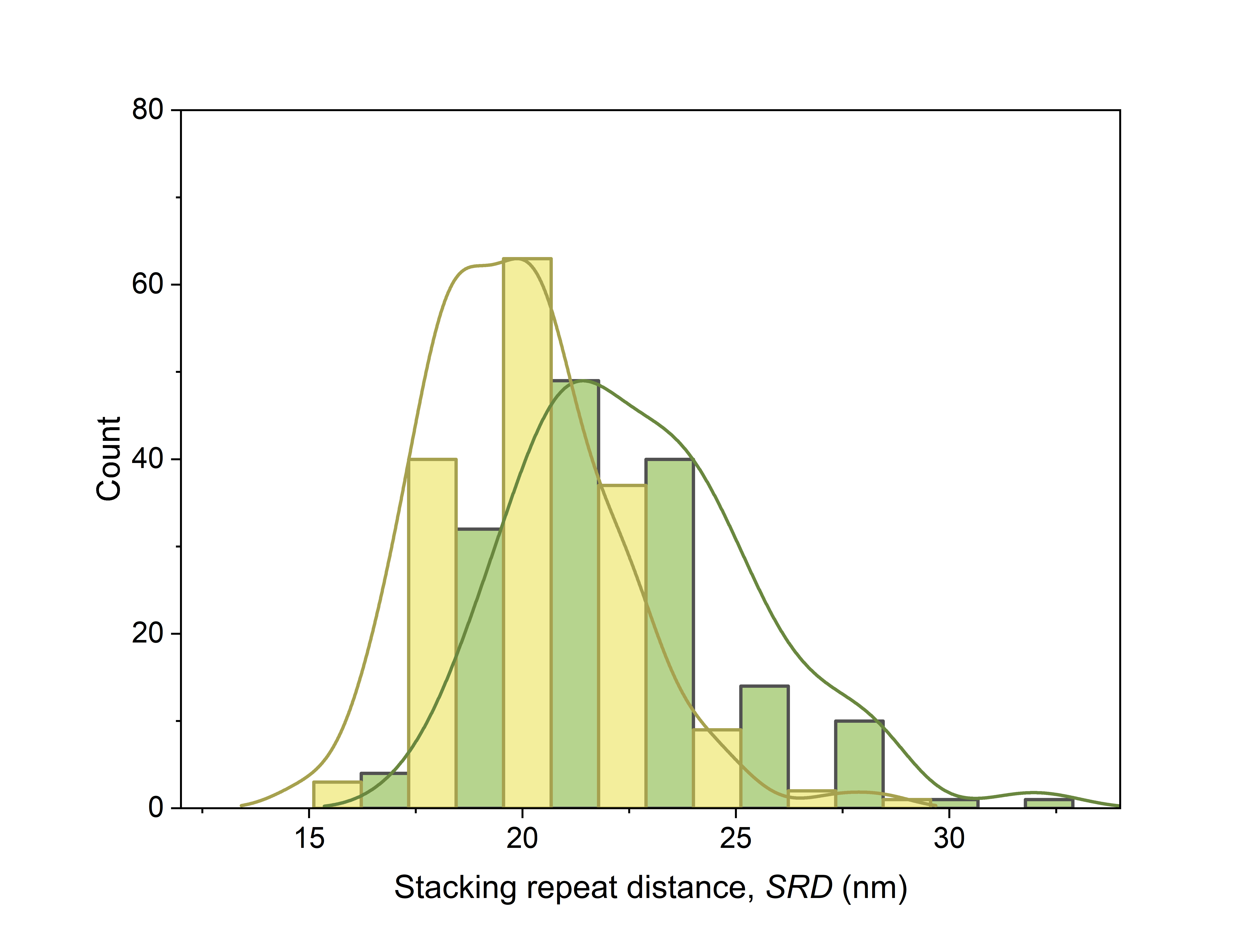


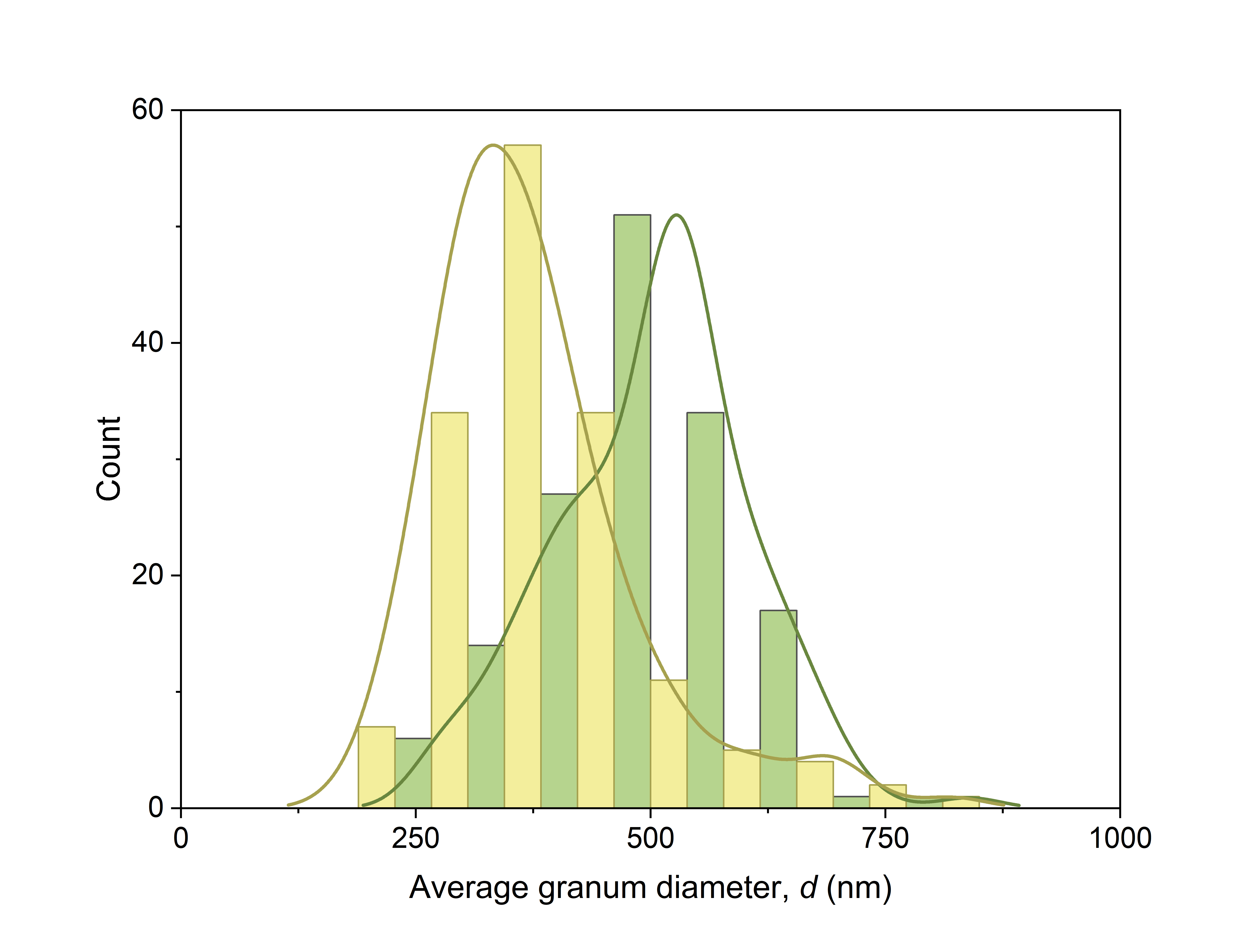

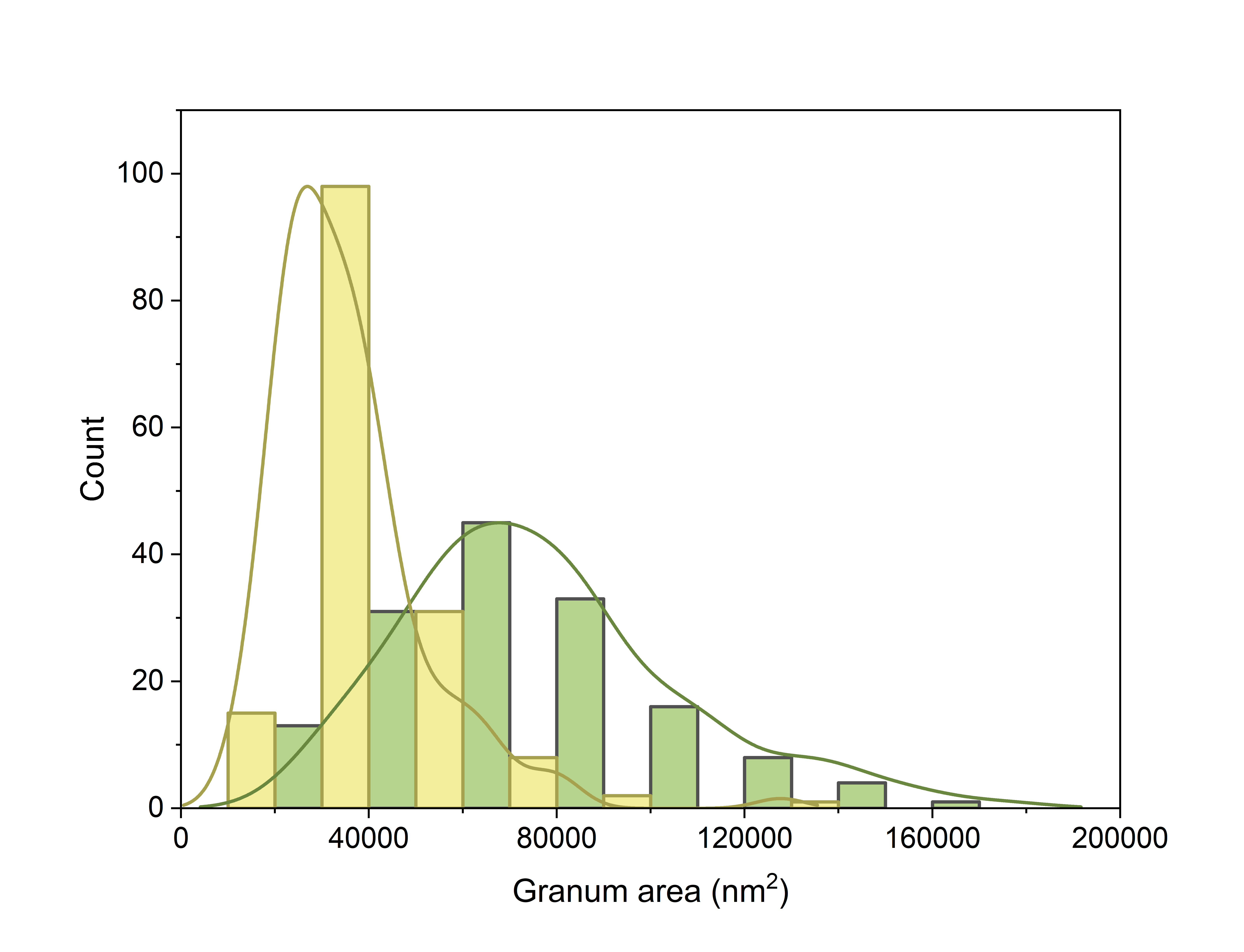


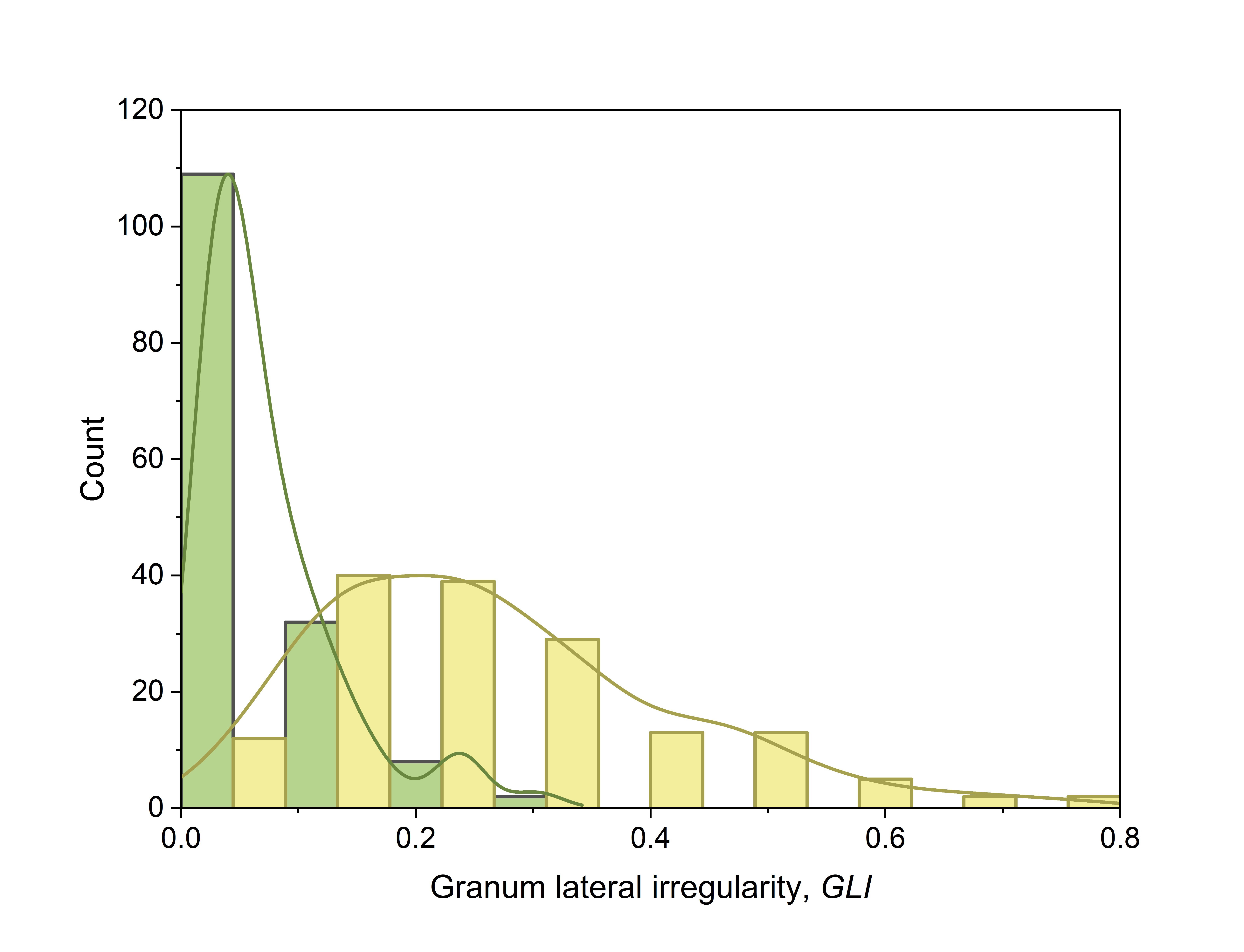

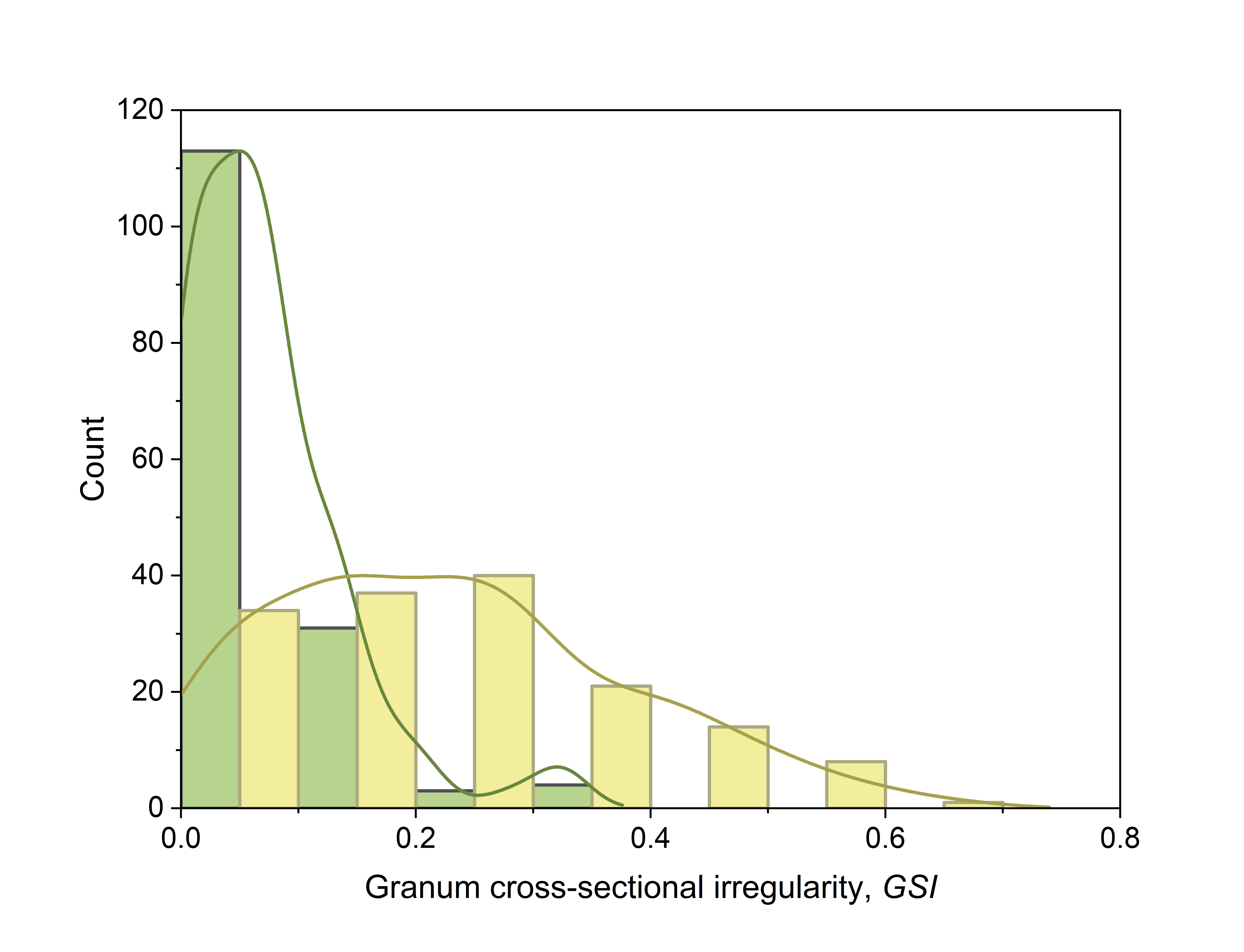

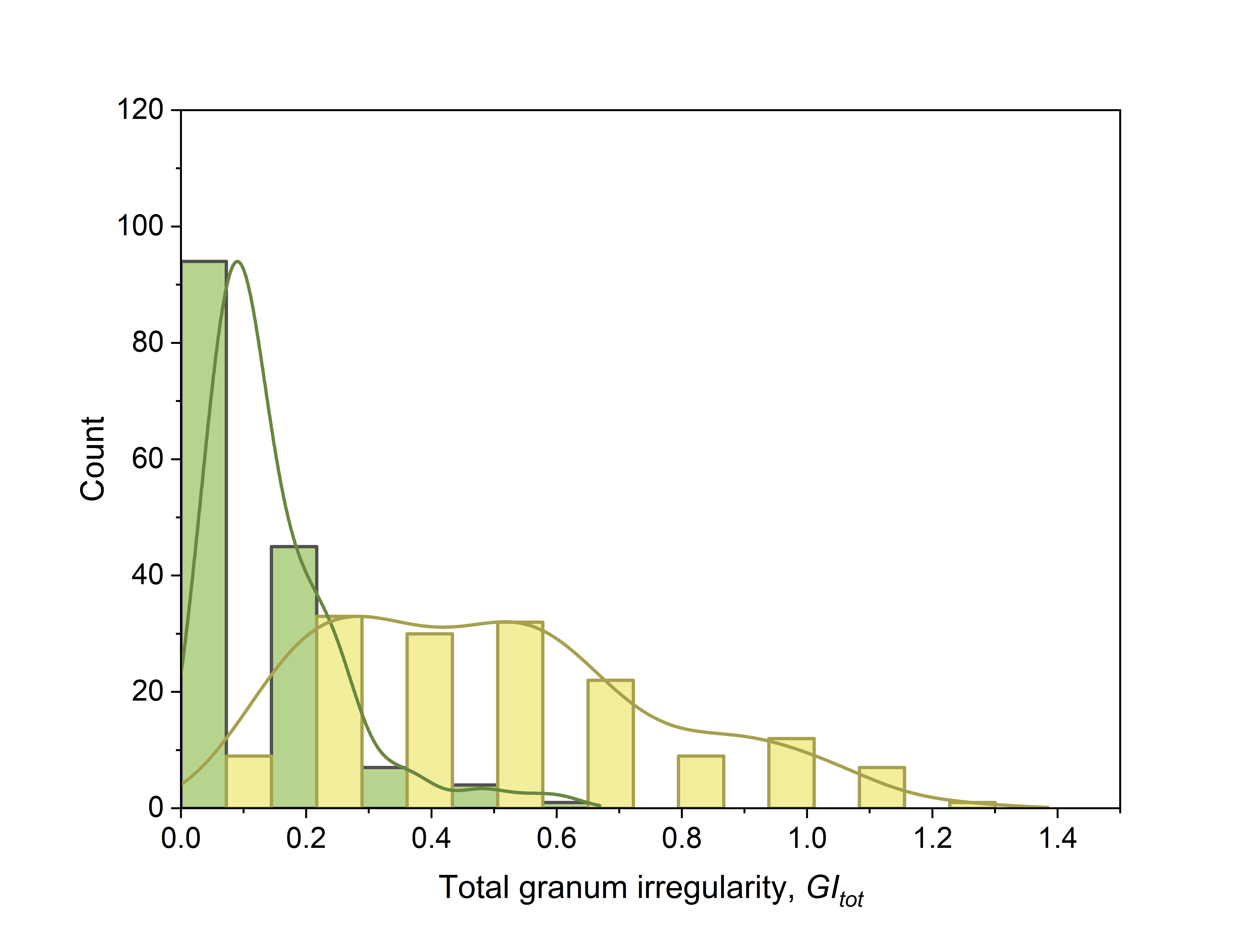


Distribution of the morphometric parameters calculated for the grana stacks in durum wheat LD222 and its chlorophyll-deficient mutant ANDW-7B. The samples size was of 151 and 155 grana, respectively. For the definition of the parameters, see the main text. In the graphs, the data distribution was approximated using the “kernel smooth” function of Origin Pro version 2024. In all cases, except *d* in LD222, the hypothesis of normality was rejected based on Shapiro-Wilk test.
